# Thioredoxin 1 promotes autophagy through transnitrosylation of Atg7 during myocardial ischemia

**DOI:** 10.1172/JCI162326

**Published:** 2023-02-01

**Authors:** Narayani Nagarajan, Shin-ichi Oka, Jihoon Nah, Changgong Wu, Peiyong Zhai, Risa Mukai, Xiaoyong Xu, Sanchita Kashyap, Chun-Yang Huang, Eun-Ah Sung, Wataru Mizushima, Allen Sam Titus, Koichiro Takayama, Youssef Mourad, Jamie Francisco, Tong Liu, Tong Chen, Hong Li, Junichi Sadoshima

**Affiliations:** 1 Department of Cell Biology and Molecular Medicine, Cardiovascular Research Institute, Rutgers New Jersey Medical School, Newark, New Jersey, USA.; 2 Center for Advanced Proteomics Research, Department of Microbiology, Biochemistry, and Molecular Genetics, Rutgers New Jersey Medical School and Cancer Institute of New Jersey, Newark, New Jersey, USA.; 3 Department of Cardiology, Ningbo Medical Center Lihuili Hospital, Ningbo, Zhejiang, China.; 4 Division of Cardiovascular Surgery, Department of Surgery, Taipei Veterans General Hospital, Taipei, Taiwan.; 5 Institute of Clinical Medicine, School of Medicine National Yang-Ming University, Taipei, Taiwan.

**Keywords:** Cardiology, Cell Biology, Autophagy, Hypoxia

## Abstract

Modification of cysteine residues by oxidative and nitrosative stress affects structure and function of proteins, thereby contributing to the pathogenesis of cardiovascular disease. Although the major function of thioredoxin 1 (Trx1) is to reduce disulfide bonds, it can also act as either a denitrosylase or transnitrosylase in a context-dependent manner. Here we show that Trx1 transnitrosylates Atg7, an E1-like enzyme, thereby stimulating autophagy. During ischemia, Trx1 was oxidized at Cys32-Cys35 of the oxidoreductase catalytic center and S-nitrosylated at Cys73. Unexpectedly, Atg7 Cys545-Cys548 reduced the disulfide bond in Trx1 at Cys32-Cys35 through thiol-disulfide exchange and this then allowed NO to be released from Cys73 in Trx1 and transferred to Atg7 at Cys402. Experiments conducted with Atg7 C402S–knockin mice showed that S-nitrosylation of Atg7 at Cys402 promotes autophagy by stimulating E1-like activity, thereby protecting the heart against ischemia. These results suggest that the thiol-disulfide exchange and the NO transfer are functionally coupled, allowing oxidized Trx1 to mediate a salutary effect during myocardial ischemia through transnitrosylation of Atg7 and stimulation of autophagy.

## Graphical abstract

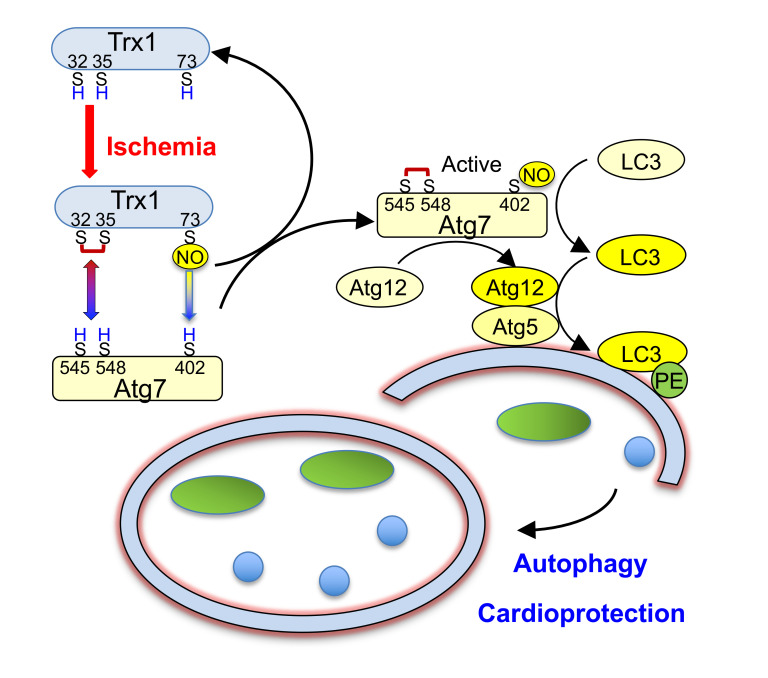

## Introduction

Thioredoxin1 (Trx1) is a 12 kDa oxidoreductase with an evolutionarily conserved CXXC motif at Cys32 and Cys35 in its catalytic center (1). Trx1 reduces proteins with disulfide bonds through thiol-disulfide exchange, in which the reduced cysteines in the catalytic center sequentially form intermolecular disulfide bonds with oxidized cysteines on their targets, thereby transferring electrons. The thiol-disulfide exchange oxidizes the catalytic cysteines in Trx1, which can then be reduced by thioredoxin reductase, using NADPH as an electron donor, to regenerate reduced Trx1 (1).

Trx1 attenuates mitochondrial dysfunction, pathological hypertrophy, and ischemic injury, thereby protecting the heart (2–7). Trx1 decreases the level of the reactive oxygen species H_2_O_2_, through reduction of peroxiredoxins. In addition, Trx1 directly interacts with signaling molecules and transcription factors, including peroxiredoxins (8), AMPK (2), and mTOR (9), and regulates their functions through reduction of disulfide bonds at cysteine residues (10).

Increasing lines of evidence suggest that Trx1 also has another important function, namely the regulation of reactive nitrogen species via modulation of protein S-nitrosylation (alternatively referred to as S-nitrosation), including transnitrosylation and denitrosylation (11). S-nitrosylation reversibly modifies cysteine residues, thereby regulating protein function (12, 13). Although proteins can be nitrosylated directly by small-molecule S-nitrosothiols (SNOs) such as S-nitrosoglutathione (GSNO), NO can also be transferred from other protein-SNOs through transnitrosylation (14). This process is highly efficient and takes place in a regulated manner at specific subcellular localizations (15, 16), thereby functioning as a defined signaling mechanism (14). Using its ability to carry SNO on its own cysteine residues, Trx1 can act as either a denitrosylase (17–19) or a transnitrosylase (20–24) and controls the level of SNO in various proteins (11). Trx1 denitrosylates cysteine residues of annexin-1, 14-3-3, and GAPDH in noncardiac cells via its authentic catalytic center Cys32-Cys35 (17–19, 24). However, Trx1 possesses a distinct catalytic center at Cys73 for protein S-nitrosylation. We and others have shown that Trx1 is S-nitrosylated at Cys69 or Cys73 (20, 22, 23) and transnitrosylates proteins, including peroxiredoxin, cyclophilin A, and caspase-3, in noncardiac cells (22–24). The molecular mechanism through which Trx1 acts as a transnitrosylase rather than a denitrosylase for some targets is not well understood. Trx1 is S-nitrosylated at Cys73 when Cys32-Cys35 forms a disulfide bond; thus, inhibition of its oxidoreductase activity promotes S-nitrosylation of Trx1 (21, 23). Conversely, S-nitrosylation of Trx1 inhibits the oxidoreductase activity of Trx1 (21). Since the major cellular functions of Trx1 are mediated primarily by the reduced form of Trx1, this raises another question as to which cellular conditions allow Trx1 to transfer NO from Cys73 to other proteins in vivo and whether this is adaptive or maladaptive.

Autophagy, a mechanism of lysosomal cellular degradation characterized by the presence of autophagosomes, is upregulated in response to ischemic preconditioning and during ischemia and protects the heart against injury (25). Autophagy is activated in stress environments, such as when there is a limited availability of nutrients during starvation and under ischemic conditions. Atg7 is a noncanonical E1-like enzyme and a key regulator of autophagy that activates Atg12 and Atg8, promoting conjugation with Atg5 and phosphatidylethanolamine, respectively (26, 27). We have shown previously that Atg7 can form an intermolecular disulfide bond with Trx1 in the heart (2), suggesting that the activity of Atg7 may be regulated by Trx1.

In the current study, we found that Trx1 is oxidized at Cys32-Cys35 and S-nitrosylated at Cys73 in response to ischemia. Furthermore, Atg7 is transnitrosylated through a transfer of NO from Trx1 Cys73 during ischemia. Based on these results, we further investigated how NO is transferred from Trx1 to Atg7 during myocardial ischemia and whether oxidized Trx1 mediates protection against myocardial ischemia. We here show that the thiol-disulfide exchange and the NO transfer are coupled, allowing oxidized Trx1 to mediate a salutary effect in the heart through transnitrosylation of Atg7 during myocardial ischemia.

## Results

### Trx1 regulates cell survival via S-nitrosylation at Cys73 during energy stress.

**Figure 1:**
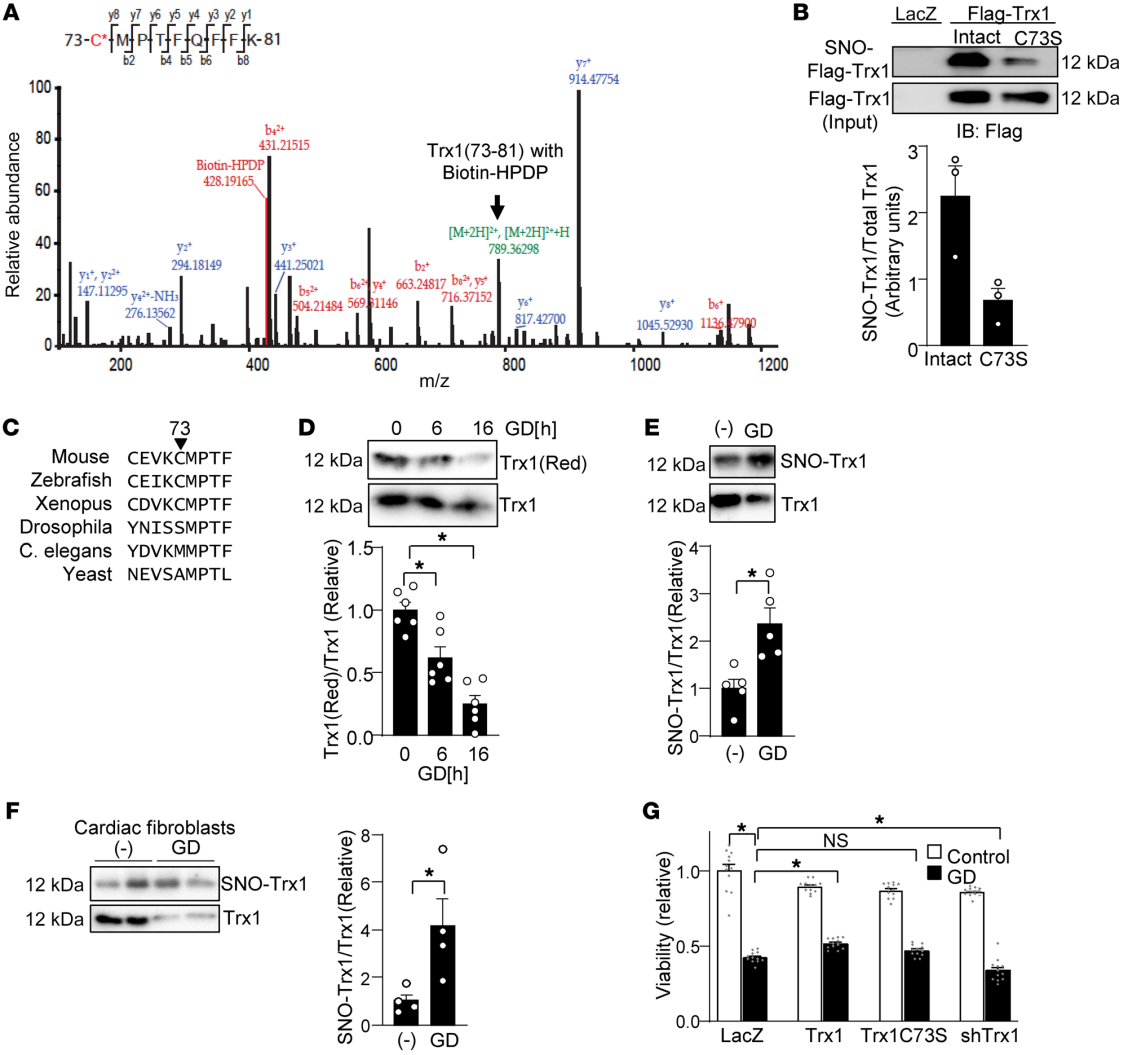
Trx1 Cys73 regulates cell survival during glucose deprivation (GD). (**A**) Recombinant Trx1 was S-nitrosylated using GSNO in vitro. The NO group was replaced with biotin-HPDP in biotin switch assays. LC-MS/MS analyses were performed. The MS/MS spectrum of *m*/*z* 789.36 resulting from a doubly charged ion (*m*/*z* 788.86) with an isotope space (0.5) corresponds to the peptide sequence of ^73^CMPTFQFFK^81^ with a biotin-HPDP (+428.19 Da) modification on Cys73 in Trx1. The b- and y-ion series correspond to the fragment ions of peptides from the N- and C-terminus, respectively, which confirms the peptide sequence of Trx1(73–81). The b-ion series (from the N-terminus), but not y-ion series (from the C-terminus), correspond to the fragment ions with a biotin-HPDP, indicating covalent binding of biotin-HPDP to Cys73. (**B**) Cys73 of Trx1 is S-nitrosylated. Cardiomyocytes were transduced with Ad-LacZ, Ad-Flag-Trx1 WT-HA, or Ad-Flag-Trx1(C73S)-HA for 48 hours. A biotin switch assay was performed, followed by pull-down with streptavidin-agarose to detect S-nitrosylated Trx1. The ratio of S-nitrosylated Trx1/total Trx1 is shown. *P < 0.05 vs. Trx1 WT, *n* = 3. Error bars represent SEM. (**C**) Cys73 is conserved among vertebrates. (**D**) Trx1 is oxidized during GD. Cardiomyocytes were lysed with biotin-labeled iodoacetamide (BIAM) after indicated periods of GD. The BIAM-labeled reduced form of Trx1 was pulled down with streptavidin-agarose. *n* = 6. (**E**) Trx1 is S-nitrosylated in response to GD in cardiomyocytes. SNO-Trx1 was detected by biotin switch assay. *n* = 5. (**F**) Trx1 is S-nitrosylated in response to GD in cardiac fibroblasts. (**G**) Cys73 of Trx1 promotes cell survival under GD conditions. Cardiomyocytes were transduced with *lacZ*, Flag-Trx1 WT-HA, Flag-Trx1(C73S)-HA, and shTrx1 adenoviruses for 48–96 hours, after which they were incubated with normal or glucose-free medium for 24 hours. Cell death was assessed using CellTiter-Blue. *n* = 12. *P < 0.05 by 2-tailed Student’s *t* test (**B**, **E**, and **F**) or 1-way ANOVA (**D** and **G**)

Previous studies showed that Trx1 is S-nitrosylated at Cys73 when the catalytic center for thiol-disulfide exchange (Cys32 and Cys35) is oxidized to form an intramolecular disulfide bond (20–24). To confirm that Cys73 in Trx1 can be S-nitrosylated, recombinant human Trx1 was treated with GSNO and subjected to a biotin switch assay, followed by trypsin digestion and mass spectrometry (MS) analyses. We verified that Cys32-Cys35 formed a disulfide bond in recombinant Trx1 (data not shown). Biotin-HDPD (*N*-[6-(biotinamide) hexyl]-3′-[2-pyridyldithiol] propionamide) labeling at C*MPTFQFYK, with C* representing the S-nitrosylated Cys residue, indicated that Trx1 is S-nitrosylated at Cys73 (Figure 1A). To further validate this result in cardiomyocytes, we transduced Ad-Flag-Trx1 WT-HA and Ad-Flag-Trx1(C73S)-HA into cardiomyocytes and evaluated the extent of Trx1 S-nitrosylation. The Trx1(C73S) mutant showed significantly less S-nitrosylation than Trx1 WT, confirming that Trx1 is S-nitrosylated at Cys73 in cardiomyocytes (Figure 1B).

We next investigated how Trx1 is S-nitrosylated at Cys73, which is evolutionarily conserved among vertebrates (Figure 1C). We hypothesized that S-nitrosylation of Trx1 at Cys73 is induced in response to glucose deprivation (GD). GD may negatively affect the production of NADPH through the pentose phosphate pathway and induce disulfide bond formation in Trx1 at the catalytic center, which should, in turn, induce S-nitrosylation of Trx1 at Cys73. To investigate whether GD oxidizes Trx1, cardiomyocytes were lysed and incubated with biotin-labeled iodoacetamide (BIAM), which covalently and irreversibly binds to reduced thiol. The BIAM-bound proteins were then recovered with streptavidin-agarose. As shown in Figure 1D, the BIAM-labeled reduced form of Trx1 was decreased after GD, suggesting that GD induces Trx1 oxidation. Biotin switch assays showed that Trx1 was more S-nitrosylated under GD conditions, consistent with our hypothesis (Figure 1E). We also investigated whether GD induces S-nitrosylation of Trx1 in cardiac fibroblasts as well. Although GD downregulated total Trx1, GD increased the S-nitrosylated Trx1/total Trx1 ratio in cardiac fibroblasts (Figure 1F).

To evaluate the functional significance of S-nitrosylation of Trx1, we transduced cardiomyocytes with adenovirus harboring Flag-Trx1 WT-HA, Flag-Trx1(C73S)-HA, or shRNA targeting Trx1 (shTrx1). Adenovirus harboring *lacZ* was used as a control. Trx1 WT decreased cardiomyocyte death after 24 hours of GD, an in vitro model of energy stress in cardiomyocytes (28). However, the protective effect was abolished with Trx1(C73S) or knockdown of Trx1 in cardiomyocytes (Figure 1G). These results suggest that the presence of Trx1 with an intact S-nitrosylation site at Cys73 promotes cell survival, whereas downregulation of endogenous Trx1 or the presence of Trx1 with a mutation at Cys73 promotes cell death during GD in cardiomyocytes. Thus, S-nitrosylation of Trx1 at Cys73 protects cardiomyocytes against cell death during GD.

### Trx1 mediates autophagy via Cys73 during energy stress.

**Figure 2:**
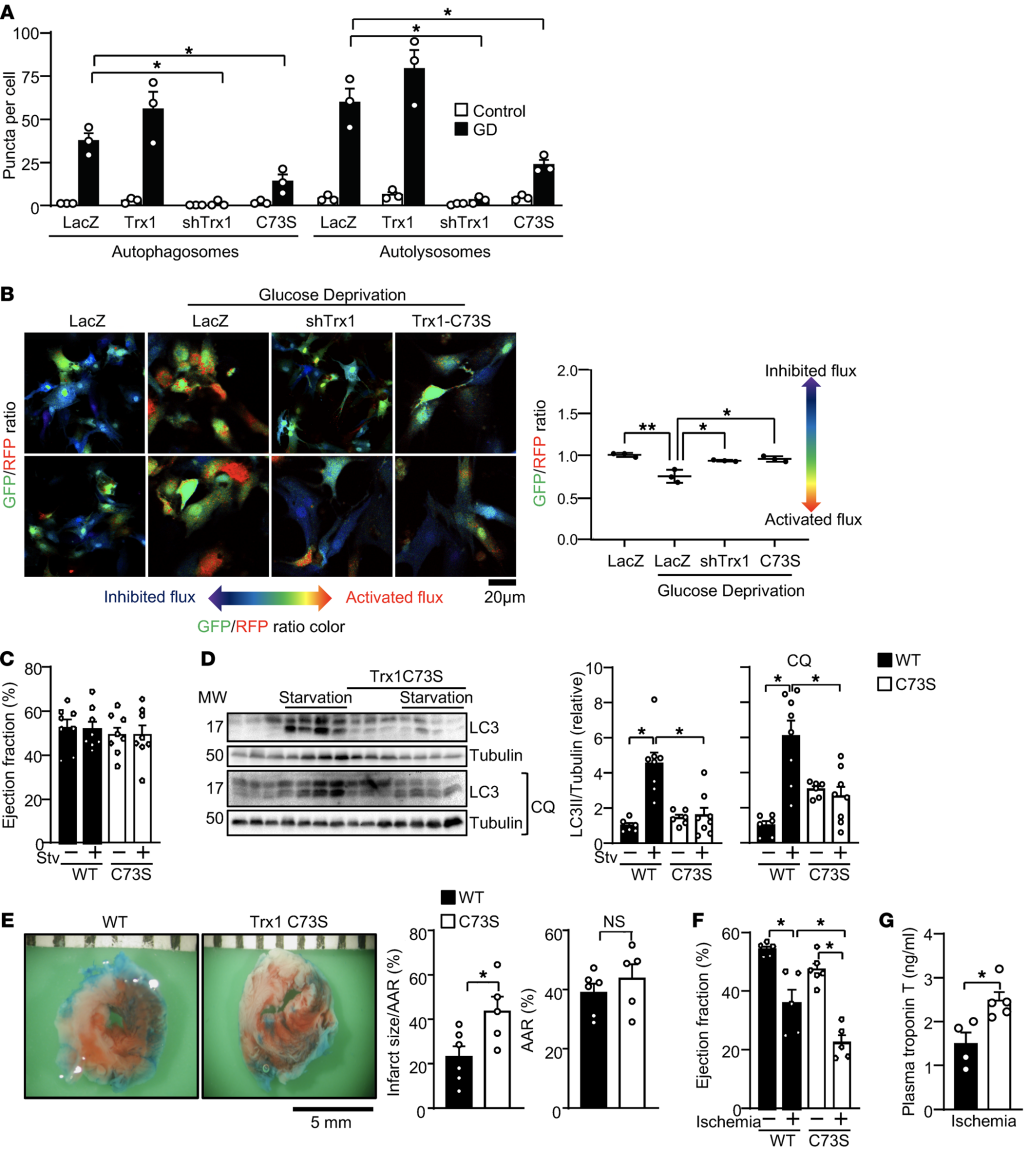
Trx1 Cys73 promotes autophagy during glucose deprivation. (**A**) Cardiomyocytes were transduced with the indicated adenovirus along with Ad-mRFP-GFP-LC3 for 48 hours. Cardiomyocytes were cultured in normal or glucose-free medium for 4 hours. Autophagosomes and autolysosomes (yellow and free red puncta) were counted and quantified. *n* = 3. (**B**) Autophagic flux was assessed using Ad-GFP-LC3-RFP. Indicated adenovirus vectors were transduced into cardiomyocytes. The cells were incubated with glucose-free medium for 4 hours. The GFP/RFP ratio is shown. *n* = 3. Scale bar: 20 μm. (**C**) Trx1-C73S–KI mice exhibited normal cardiac function under basal and starvation conditions. *n* = 8. (**D**) Trx1 Cys73 mediates starvation-induced autophagy in the heart. LC3-II formation was evaluated after 48 hours of starvation with chloroquine (CQ) treatment. *n* = 6–8. (**E**) Trx1-C73S–KI mice and WT mice were subjected to ischemia for 3 hours. Representative images of TTC staining are shown. Scale bar: 5 mm. The percentage infarct area/area at risk (AAR) and AAR (%) are shown. (**F**) Echocardiographic measurements after 3 hours of ischemia. *n* = 5–6. (**G**) Plasma troponin T levels were evaluated after 3 hours of ischemia. *n* = 4–5. Error bars represent SEM. *P < 0.05; **P < 0.01 by 1-way ANOVA (**A**–**D** and **F**) or 2-tailed Student’s *t* test (**E** and **G**).

Since the cell protective effect mediated by Trx1 Cys73 is observed during GD, a condition in which autophagy is activated in cardiomyocytes (28), we went on to study whether Trx1 affects autophagy. To this end, cardiomyocytes were subjected to GD together with either upregulation or downregulation of Trx1 or in the presence of Trx1(C73S). As shown in Figure 2A, after 4 hours of GD, autophagy was evaluated using tandem fluorescent LC3 (mRFP-GFP-LC3) (29). As previously reported, GD increased the numbers of both autophagosomes, labeled with both GFP and mRFP, and autolysosomes, labeled with mRFP only, indicating increased autophagic flux (29). The numbers of autophagosomes and autolysosomes induced by GD both tended to be increased in the presence of WT Trx1, but this did not reach statistical significance. On the other hand, GD-induced increases in autophagosomes and autolysosomes were significantly inhibited in the presence of either shTrx1 or Trx1(C73S). Representative microscopic images are shown in Supplemental Figure 1A; supplemental material available online with this article; https://doi.org/10.1172/JCI162326DS1 Autophagic flux was also evaluated with GFP-LC3-RFP (30). In this system, GFP-LC3-RFP is cleaved by endogenous Atg4B into GFP-LC3 and RFP. GFP-LC3 is degraded by autophagy, while RFP is not, serving as an internal control; thus, autophagic flux can be estimated by measuring the GFP/RFP signal ratio. Blue (computer-generated color representing high GFP/RFP) indicates low flux, whereas yellow/orange (representing low GFP/RFP) indicates high flux. Consistent with our previous results, we found that Trx1 knockdown and Trx1(C73S) attenuated GD-induced increases in autophagic flux in cardiomyocytes (Figure 2B). Low-power images are shown in Supplemental Figure 1B. Taken together, these data suggest that either endogenous Trx1 or a Trx1 Cys73–dependent mechanism plays an important role in mediating autophagy during GD in cardiomyocytes.

To test whether Trx1 mediates autophagy via a Cys73-dependent mechanism in the heart, we generated Trx1 C73S–knockin (Trx1-C73S–KI) mice. Echocardiography showed that Trx1-C73S–KI mice exhibited normal cardiac dimensions and left ventricular contractile function both under basal conditions and after 48 hours of fasting (Figure 2C and Supplemental Table 1). However, despite normal cardiac function during starvation, starvation-induced autophagy, evidenced by LC3-II formation, was inhibited in Trx1-C73S–KI mice in both the presence and absence of chloroquine (Figure 2D). Furthermore, after 3 hours of myocardial ischemia, Trx1-C73S–KI mice exhibited larger myocardial infarcts, as evaluated with tetrazolium chloride (TTC) staining (Figure 2E), decreases in ejection fraction, an index of cardiac contractile function evaluated by echocardiography (Figure 2F and Supplemental Table 2), and increases in plasma troponin T levels, an index of cardiomyocyte injury, compared with WT mice (Figure 2G). These results suggest that a Cys73-dependent mechanism protects the heart against ischemia.

### Trx1 and Atg7 undergo thiol-disulfide exchange.

**Figure 3:**
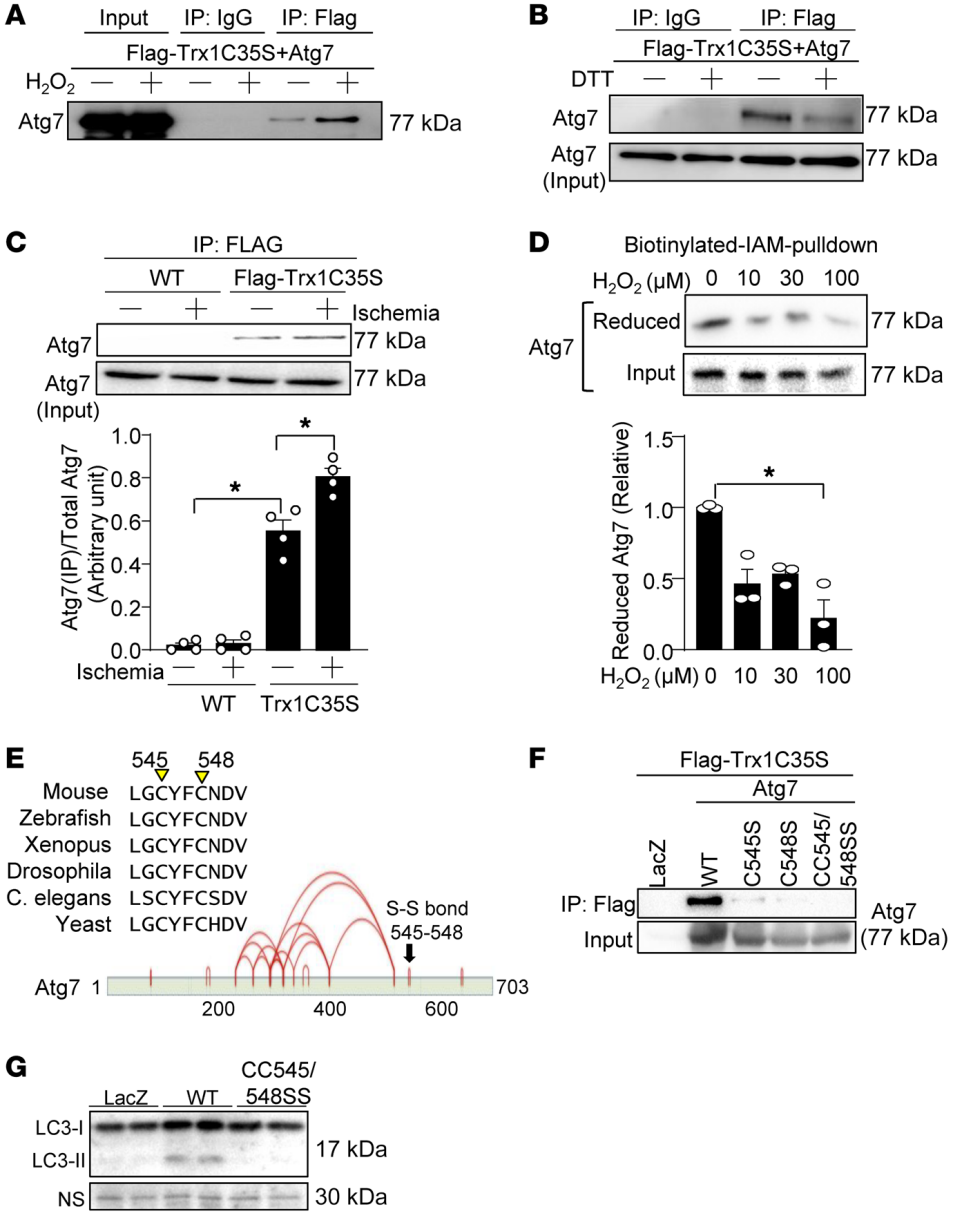
Trx1 and Atg7 interact via disulfide linkage, which is enhanced during oxidative/energy stress. (**A**) Cardiomyocytes were transduced with Ad-Flag-Trx1 C35S and Ad-Atg7 for 48 hours and then treated with 100 μM H_2_O_2_ for 30 minutes. Interaction between Trx1 and Atg7 was examined. (**B**) Cardiomyocytes were transduced with Ad-Flag-Trx1 C35S and Ad-Atg7 for 48 hours and then interaction between Trx1 and Atg7 was examined in the presence or absence of DTT. (**C**) Tg-Flag-Trx1 C35S and WT mice were subjected to sham operation or ischemia for 20 minutes. Homogenates were prepared from sham/ischemic areas. Coimmunoprecipitation with anti-FLAG–agarose beads followed by immunoblotting for Atg7 was performed. Representative immunoblots are shown. *n* = 4. (**D**) Cardiomyocytes were treated with the indicated concentrations of H_2_O_2_ for 10 minutes and labeled with biotin-labeled iodoacetamide (BIAM) upon lysis. Atg7 with reduced cysteines was recovered with streptavidin-agarose. *n* = 3. *P < 0.05 by 1-way ANOVA (**C**) or Kruskal-Wallis test (**D**). (**E**) Evolutionarily conserved Cys545 and Cys548 form an intramolecular disulfide bond. Intramolecular disulfide bonds were identified by MS analysis using recombinant Atg7. Evolutionary conservation of Cys545 and Cys548 is shown (upper panel). Intramolecular disulfide bonds are indicated by red lines (lower panel). (**F**) HEK293 cells were transduced with the indicated plasmids for 48 hours and then interaction between Trx1 and Atg7 was examined. (**G**) Atg7-KO AMCMs were transduced with Ad-LacZ, Ad-Atg7 WT, or Ad-Atg7 CC545/548SS for 24 hours. Protein samples were prepared and Western blotting was performed to detect LC3 and Atg7.

To understand the mechanism by which Trx1 regulates autophagy, we screened for key autophagy proteins that interact with Trx1, using a trapping-mutant strategy (2, 10). In this system, the Trx1 trapping mutant, in which Cys35 is mutated to Ser, forms a stable intermolecular disulfide bond between Cys32 and cysteine residues in target proteins, allowing the target proteins to be isolated by coimmunoprecipitation (co-IP) with the Trx1 trapping mutant. We reported previously that the Trx1 trapping mutant interacts with Atg7 in the mouse heart at baseline in vivo (2). Although the antibody array we used to screen Trx1 targets also included other known regulators of autophagy, including Atg4B, Atg5, Beclin1, p62, ULK1, UVRAG, and VPS34, they did not interact with the Trx1 trapping mutant (2). We therefore focused on Atg7 here in investigating the mechanism by which Trx1 regulates autophagy. Cardiomyocytes were transduced with adenovirus harboring the Trx1 trapping mutant, Ad-Flag-Trx1 C35S-HA, and protein samples were subjected to IP with anti-FLAG–agarose beads. Atg7 was pulled down with the anti-FLAG–agarose beads, a result that was significantly enhanced when the cardiomyocytes were treated with 100 μM H_2_O_2_ for 30 minutes (Figure 3A). The interaction between Trx1 C35S and Atg7 was markedly decreased in the presence of dithiothreitol (DTT), a reducing agent that breaks down disulfide bonds, suggesting that the interaction is due to a disulfide bond (Figure 3B).

To verify the results in vivo, the interaction between Trx1 and Atg7 was analyzed under sham and ischemic conditions in transgenic mice expressing FLAG-Trx1 C35S-HA in a cardiac-specific manner (2) (trapping mice). Atg7 was pulled down with an anti-FLAG antibody from FLAG-Trx1C35S–transgenic, but not WT, mice. FLAG-Trx1C35S interacted with Atg7 in the sham-operated group, and this interaction was strengthened significantly following 20 minutes of ischemia (Figure 3C). These results suggest that oxidative stress promotes Trx1-Atg7 interaction both in vitro and in vivo.

To further confirm that one or more cysteine residues in Atg7 is redox sensitive, we performed BIAM pulldown assays. In cardiomyocytes, treatment with H_2_O_2_ for 10 minutes dose-dependently reduced labeling of Atg7 with BIAM, suggesting that cysteine residues in Atg7 can be oxidized by H_2_O_2_ (Figure 3D). Next, we mapped the cysteine residues in Atg7 that are involved in disulfide bond formation with Trx1. As a first step, we identified reactive cysteine residues in Atg7, using liquid chromatography–tandem MS (LC-MS/MS). Cardiomyocytes were treated with H_2_O_2_ for 10 minutes and, after homogenization, the reduced cysteine residues were irreversibly marked with iodoacetamide while the redox-sensitive cysteine residues were labeled with S-methyl methanethiosulfate (MMTS) after reduction with tris(2-carboxyethyl)phosphine hydrochloride. Endogenous rat Atg7 was immunoprecipitated and subjected to MS analyses. The MS/MS spectra indicated that several reactive cysteine residues in Atg7, including Cys294, -354, -364, -402, -545, and -548, were labeled with MMTS. Since rat Atg7 Cys545 and Cys548 are evolutionarily conserved and located in a consensus sequence for the active site for thiol oxidoreductase (CXXC) (Figure 3E), we further investigated whether these cysteine residues form disulfide bond(s). MS analysis using recombinant human Atg7 identified multiple disulfide bonds, including Cys550 and Cys553, corresponding to Cys545 and Cys548 in mouse and rat Atg7 (Figure 3E). To test whether Cys545 and Cys548 are targets of Trx1, we made the following mutants of mouse Atg7: Atg7(C545S), Atg7(C548S), and Atg7(C545S/C548S). When these mutants were coexpressed with the Trx1 trapping mutant in cardiomyocytes, they all exhibited a reduced interaction with Trx1 compared with WT Atg7 (Figure 3F). Thus, Trx1 interacts with Atg7 via Cys545-Cys548, possibly through thiol-disulfide exchange. To test whether Cys545-Cys548 of Atg7 is required for Atg7 function, Atg7 and Atg7(C545S/C548S) were transduced into adult mouse cardiomyocytes (AMCMs) isolated from Atg7 cardiac-specific knockout (KO) mice. Importantly, there was significantly less LC3-II conversion in AMCMs reexpressing Atg7(C545S/C548S) than in those reexpressing WT Atg7 (Figure 3G). These results suggest that Trx1 interacts with Atg7 at Cys545-Cys548 and that the interaction is essential for stimulation of autophagy by Atg7.

### Trx1 oxidizes Atg7 in response to energy stress.

**Figure 4:**
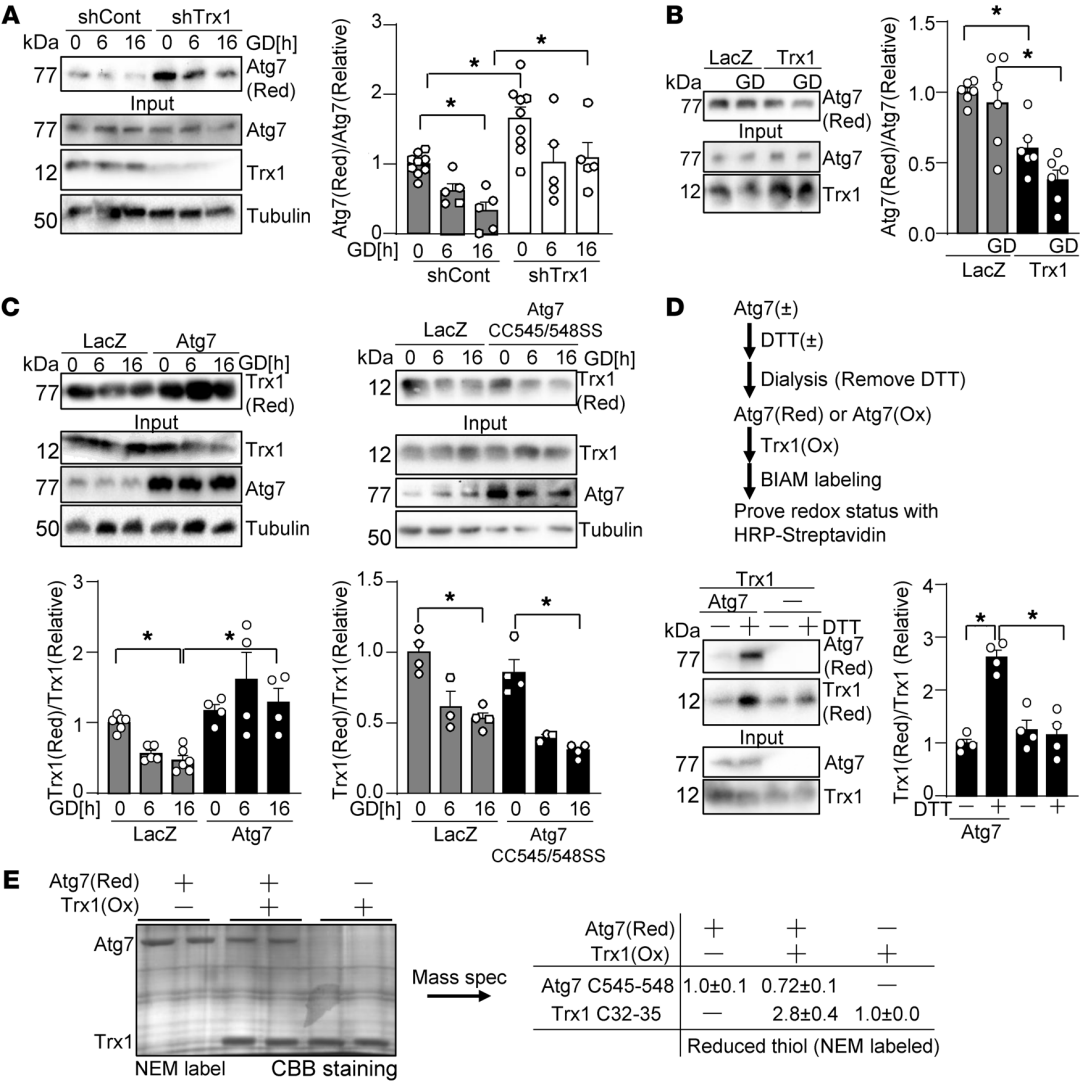
Trx1 oxidizes Atg7 during energy stress. (**A** and **B**) Trx1 promotes GD-induced Atg7 oxidation. Cardiomyocytes were transduced with Ad-shTrx1 (**A**) or Ad-Trx1 (**B**). The redox status of Atg7 was examined with BIAM pulldown. *n* = 6–10 (**A**) and 6 (**B**). (**C**) Atg7 prevents GD-induced Trx1 oxidation in a Cys545-Cys548–dependent manner. Cardiomyocytes were transduced with Ad-Atg7 or Ad-Atg7(CC545/548SS). The redox status of Trx1 was examined with BIAM pulldown. *n* = 3–6. (**D** and **E**) Atg7 reduces Trx1 in an in vitro reconstitution system. (**D**) Recombinant Atg7 was reduced with DTT and dialyzed to remove DTT. Reduced Atg7 was incubated with oxidized Trx1. Reduced Trx1 was detected with BIAM labeling. *n* = 5. (**E**) The redox status of Atg7 at Cys545-Cys548 and Trx1 at Cys32-Cys35 was examined with MS analyses. Experiments were conducted in duplicate. CBB, Coomassie brilliant blue. *P < 0.05 by 1-way ANOVA (**A**–**D**).

Atg7 possesses multiple cysteine residues that undergo oxidation (31), and, thus, could be reduced by Trx1. We evaluated the effect of Trx1 on the oxidation status of Atg7. Contrary to our expectation, knockdown of Trx1 decreased the level of Atg7 oxidation under both basal and GD conditions (Figure 4A). Conversely, overexpression of Trx1 promoted Atg7 oxidation under both basal and GD conditions (Figure 4B). Thus, contrary to the general belief, Trx1 oxidizes Atg7. In order to test whether Atg7 reduces Trx1 in a Cys545-Cys548–dependent manner, the redox status of Trx1 was evaluated. Overexpression of Atg7 prevented GD-induced oxidation of Trx1, an effect that was abolished in the presence of an Atg7-CC545/548SS mutant (Figure 4C). These results suggest that GD induces oxidation of Trx1, which is alleviated in the presence of Atg7 with intact Cys545 and Cys548. To test whether Atg7 directly reduces Trx1 in vitro, we performed in vitro redox reactions using recombinant proteins. As shown in Figure 4D, recombinant Atg7 was first reduced by DTT and then incubated with oxidized Trx1. The extent of Trx1 cysteine oxidation was evaluated with BIAM labeling. Incubation with reduced Atg7, but not oxidized Atg7, increased BIAM labeling of Trx1, suggesting that Trx1 was reduced by Atg7. To verify that thiol-disulfide exchange takes place between Cys545-Cys548 of Atg7 and Cys32-Cys35 of Trx1, MS analyses were performed. Incubation of reduced Atg7 with oxidized Trx1 resulted in an increase in disulfide bond formation in Atg7 at Cys545-Cys548 but a decrease in disulfide bond formation in Trx1 at Cys32-Cys35 (Figure 4E). These results suggest that Atg7 may reduce Trx1 at Cys32-Cys35 under GD conditions through a thiol-disulfide exchange between Atg7 Cys545-Cys548 and Trx1 Cys32-Cys35. Since previous studies have suggested that disulfide reduction facilitates denitrosylation of S-nitrosylated Trx1 (32), our results suggest that Atg7-mediated reduction of oxidized Trx1 may catalyze transnitrosylation of targets.

### Trx1 transnitrosylates Atg7 in response to energy stress.

**Figure 5:**
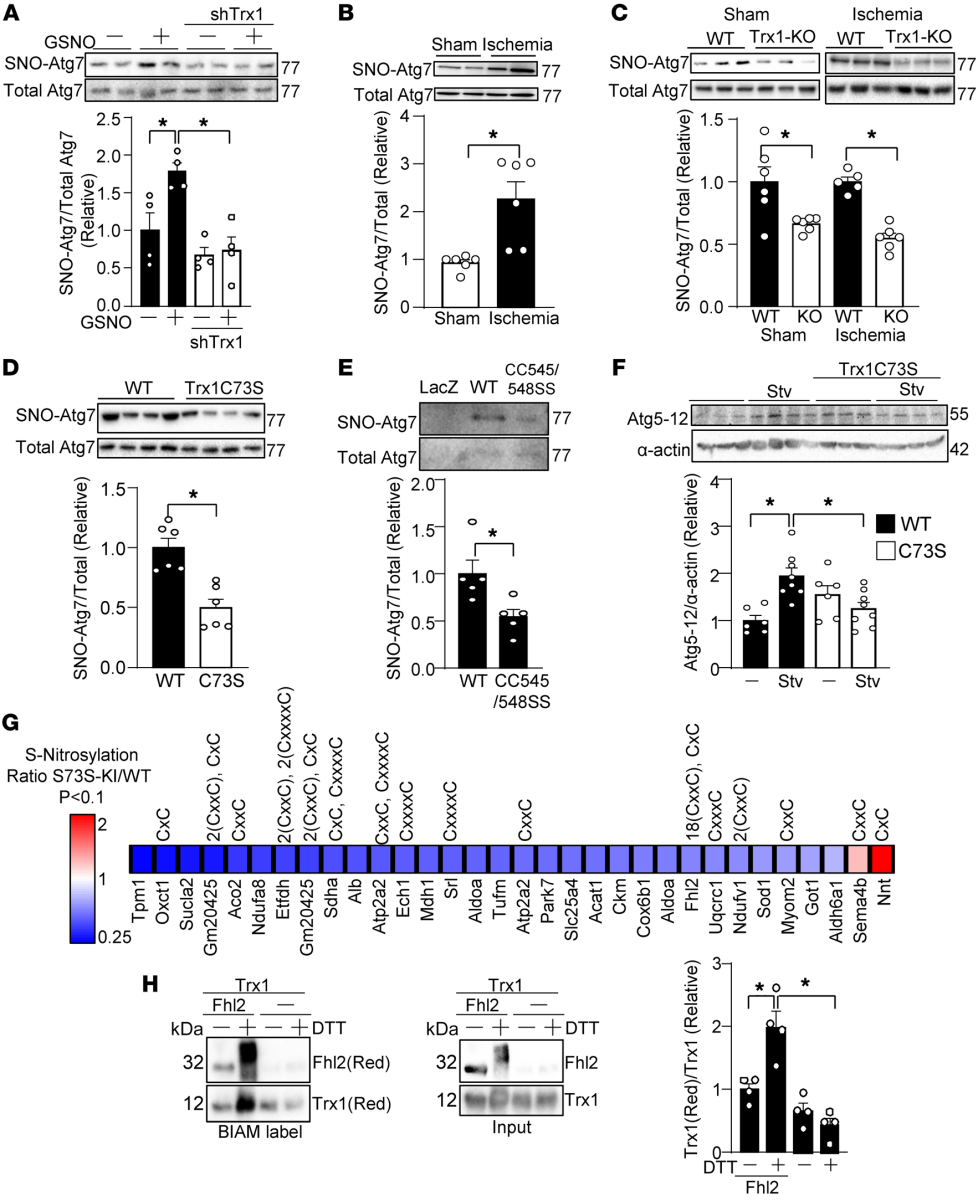
Trx1 S-nitrosylates Atg7, a process that is promoted by energy stress. (**A**–**E**) S-nitrosylation of Atg7 was analyzed with biotin switch assays. (**A**) Trx1 mediates GSNO-induced S-nitrosylation of Atg7. Cardiomyocytes were treated with 100 μM GSNO for 30 minutes. *n* = 4. (**B**) WT mice were subjected to either sham operation or ischemia for 30 minutes. *n* = 6. (**C**) WT and cardiac-specific heterozygous Trx1-KO mice were subjected to ischemia for 30 minutes. *n* = 5–6. (**D**) The level of SNO-Atg7 was examined in homozygous Trx1-C73S–KI mice. *n* = 6. (**E**) Atg7-KO AMCMs were transduced with Ad-LacZ, Ad-Atg7 WT, or Ad-Atg7 CC545/548SS for 24 hours. Biotin switch assays were performed to detect SNO-Atg7. *n* = 5. (**F**) Trx1 regulates Atg7 in a Cys73-dependent manner. Conjugation of Atg5 and Atg12 was assessed in Trx1-C73S–KI mice after 48 hours of starvation (Stv). *n* = 6. (**G**) Possible S-nitrosylation substrates of Trx1 in the heart. Proteins whose S-nitrosylation is changed in Trx1-C73S–KI mice were identified with biotin switch assays followed by MS identification. (**H**) Fhl2 reduces Trx1 in an in vitro reconstitution system. Recombinant His-tagged Fhl2 was immobilized with Ni-NTA resin and reduced with DTT. To remove DTT, Ni-NTA resin was washed with 1 mL buffer twice. Fhl2 was then incubated with oxidized Trx1. Reduced Fhl2 and Trx1 were labeled with BIAM. *n* = 4. *P < 0.05 by 1-way ANOVA (**A**, **C**, **D**, **F**, and **H**) or 2-tailed Student’s *t* test (**B**, **E**, and **G**).

Since the regulation of autophagy by Trx1 is dependent on Cys73, a residue involved in S-nitrosylation, we hypothesized that Trx1 regulation of autophagy is mediated through S-nitrosylation. Since Trx1 and Atg7 interact with one another through thiol-disulfide exchange, we hypothesized that Trx1 transnitrosylates Atg7 during energy stress. Indeed, knockdown of Trx1 inhibited GS-NO–induced S-nitrosylation of Atg7 in cardiomyocytes (Figure 5A). Next, we investigated whether Atg7 is S-nitrosylated in response to stress such as ischemia in the heart. To this end, mice were subjected to myocardial ischemia through coronary ligation and then biotin switch assays were conducted with heart homogenates. Ischemia did not change the protein level of Atg7. However, S-nitrosylation of Atg7 was significantly increased after 30 minutes of myocardial ischemia (Figure 5B). S-nitrosylation of Atg7 was reduced significantly in cardiac-specific heterozygous Trx1-KO mice under both basal and ischemic conditions (Figure 5C). These results suggest the involvement of endogenous Trx1 in S-nitrosylation of Atg7. Furthermore, there was significantly less SNO-Atg7 in Trx1-C73S–KI mice than in WT mice (Figure 5D). These results suggest that Trx1 S-nitrosylates Atg7 in a Cys73-dependent manner.

Physical interaction between the NO donor and acceptor promotes transnitrosylation (33). We thus hypothesized that physical interaction between Trx1 and Atg7 through thiol-disulfide exchange is required for Atg7 nitrosylation. Since Cys545 and Cys548 in Atg7 are involved in the thiol-disulfide exchange with Trx1, Atg7, and Atg7(CC545/548SS), a mutant that cannot interact with Trx1, were transduced into AMCMs isolated from cardiac-specific Atg7-KO mice. Biotin switch assays indicated that transnitrosylation of Atg7(CC545/548SS) was attenuated compared with that of WT Atg7 (Figure 5E). These results are consistent with the notion that interaction between Trx1 and Atg7 Cys545/Cys548 through a thiol-disulfide exchange is essential for mediating transnitrosylation of Atg7.

To determine whether Trx1 regulates Atg7 in a Cys73-dependent manner, the E1-like enzyme activity of Atg7 was evaluated based on its ability to promote Atg5-Atg12 conjugation. Starvation-induced Atg5-Atg12 complex formation was partly inhibited in Trx1-C73S–KI mice, suggesting that Trx1 potentiates Atg7 function during starvation in a Cys73-dependent manner in vivo (Figure 5F).

In order to evaluate the role of Cys73 of Trx1 in mediating transnitrosylation of cardiac proteins during myocardial ischemia, we conducted proteomics analyses using tandem mass tag (TMT) labeling of S-nitrosylated proteins in WT and Trx1-C73S–KI mice. As shown in Figure 5G, we identified 28 proteins showing decreased S-nitrosylation in Trx1-C73S–KI mice compared with those in WT mice and 2 proteins that showed increased S-nitrosylation. This suggests that Cys73 mediates transnitrosylation more frequently than denitrosylation. Of note, Atg7 was not detected in the screening, most likely because Atg7 is less abundant than the proteins listed in Figure 5G. Interestingly, approximately half of the identified proteins possess a thioredoxin-like motif such as CXC or CXXC, which may allow thiol-disulfide exchange with Trx1. Thus, we tested whether a transnitrosylation target of Trx1 other than Atg7 can reduce Trx1. To this end, we evaluated the ability of Fhl2 to reduce Trx1, since Fhl2 has multiple CXXC motifs. Recombinant Fhl2 was first reduced by DTT. After removal of DTT, reduced Fhl2 was incubated with oxidized Trx1. Trx1 was reduced in the presence of reduced Fhl2 but not vehicle alone (Figure 5H). Of note, a shift in the molecular weight of Fhl2 after DTT treatment reflects multiple BIAM labeling. These results are consistent with the notion that Trx1 transnitrosylates targets during thiol-disulfide exchange and that Trx1 may be reduced by its S-nitrosylation targets.

### Trx1 transnitrosylates Atg7 at Cys294 and Cys402.

**Figure 6:**
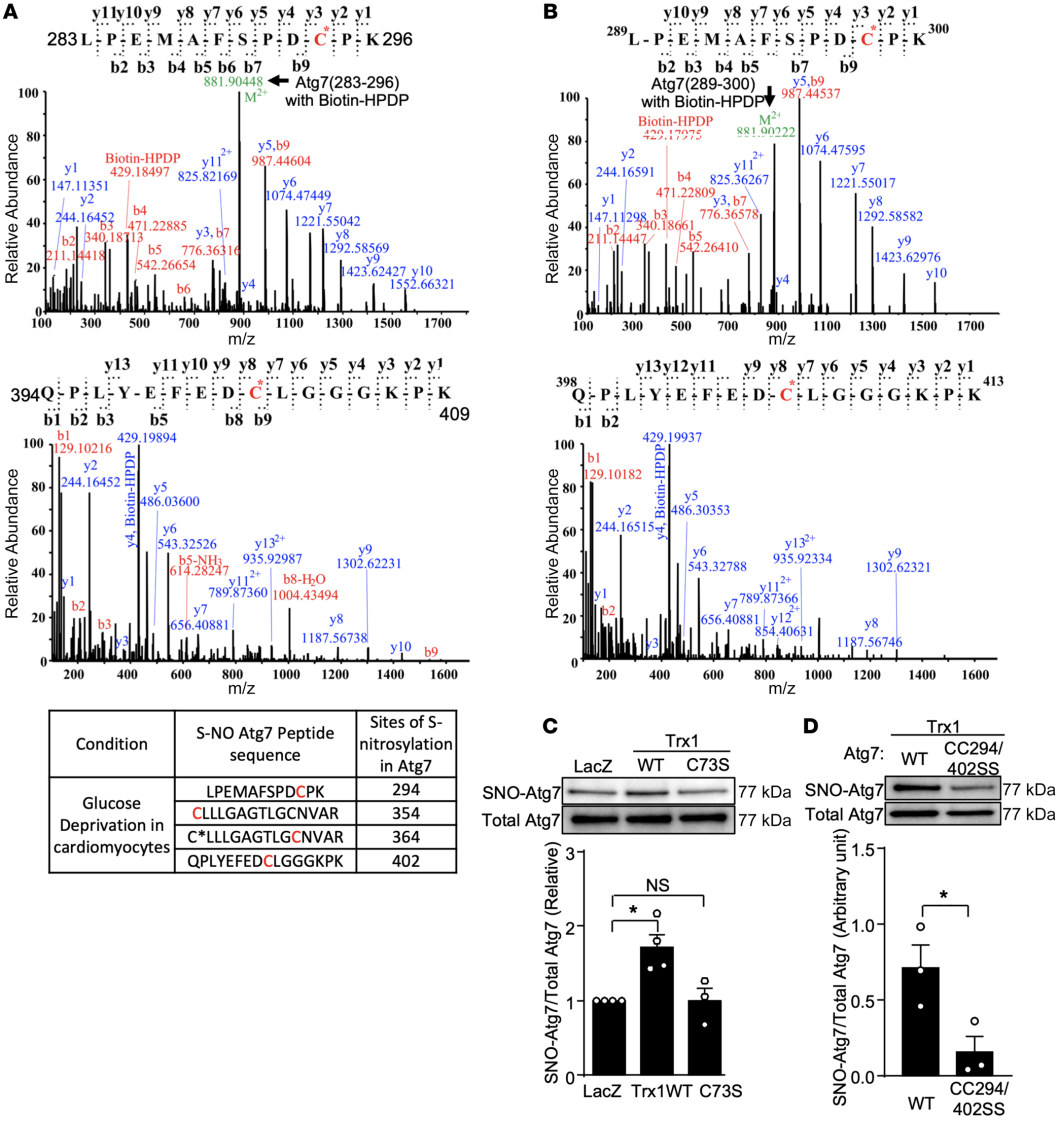
Trx1 transnitrosylates Atg7 at Cys294 and Cys402. (**A**) Rat cardiomyocytes transduced with Ad-Atg7 were cultured in normal or glucose-free medium for 4 hours. A biotin switch assay was performed, followed by MS analysis to determine sites of S-nitrosylation in Atg7. (**B**) Recombinant Trx1 treated with GSNO was incubated with recombinant human Atg7 protein for 30 minutes. A biotin switch assay followed by MS analysis was performed to determine sites of transnitrosylation in Atg7. A representative MS spectrum is shown. (**A** and **B**) The MS/MS spectra of the peptides ^283^LPEMAFSPDC*PK^296^ and ^394^QPLYEFEDC*LGGGKPK^409^ with a biotin-HPDP (+428.19 Da) modification on C294 and C402 are shown. The MS/MS spectrum of *m*/*z* 881.9 corresponds to the peptide sequence of Atg7 (283–296) with a biotin-HPDP. That of Atg7 (394–409) with a biotin-HPDP was not detected due to complete fragmentation of the precursors. The y- and b-ion series confirmed the peptide sequence and the biotin-HPDP modification on the cysteines. The rat Atg7 peptides 283–296 and 394–409 correspond to human Atg7 289–300 and 398–413, respectively. (**C**) Cardiomyocytes were transduced with Ad-LacZ, Ad-Trx1 WT, or Ad-Trx1(C73S). A biotin switch assay followed by Western blotting was carried out to measure S-nitrosylation of Atg7. Representative immunoblots and quantification analysis of S-NO Atg7/total Atg7 are shown. *n* = 3. (**D**) Cardiomyocytes were transduced with Ad-Atg7 WT or Ad-Atg7 CC294/402SS in the presence of Ad-LacZ or Ad-Trx1 WT. A biotin switch assay followed by Western blotting was performed to measure S-nitrosylation of Atg7. Representative immunoblots and quantification analysis of SNO-Atg7/total Atg7 are shown. *n* = 3. *P < 0.05 by Kruskal-Wallis test (**C**) or 2-tailed Student’s *t* test (**D**).

To identify the S-nitrosylation sites in Atg7, cardiomyocytes overexpressing Atg7 were subjected to either 4 hours of GD or normal culture conditions. The samples were then subjected to biotin switch assays, followed by trypsin digestion and MS/MS analyses. We found that Cys294, Cys354, Cys364, and Cys402 in mouse Atg7 are S-nitrosylated in response to GD (Figure 6A). We next investigated whether these cysteine residues are directly transnitrosylated by Trx1 using an in vitro reconstitution system. To this end, recombinant human Trx1 protein was incubated with GSNO and S-nitrosylated Trx1 was generated. After excess GSNO was removed by acetone precipitation, S-nitrosylated Trx1 was incubated with recombinant human Atg7 protein. The S-nitrosylation status of Atg7 was evaluated with biotin switch assays followed by LC-MS/MS analyses. Human Atg7 protein was S-nitrosylated at Cys298 and Cys406 (corresponding to Cys294 and Cys402 in mouse Atg7, respectively) in the presence of S-nitrosylated Trx1 in the in vitro reconstitution system (Figure 6B). Overexpression of Trx1 promoted S-nitrosylation of Atg7 in a Cys73-dependent manner (Figure 6C). Trx1-induced S-nitrosylation of Atg7 was inhibited by CC294/402SS mutation of Atg7 (Figure 6D). These results suggest that Trx1 transnitrosylates Atg7 at Cys294 and Cys402 in a Cys73-dependent manner.

### S-nitrosylation of Atg7 promotes E1-like activity and autophagy.

**Figure 7:**
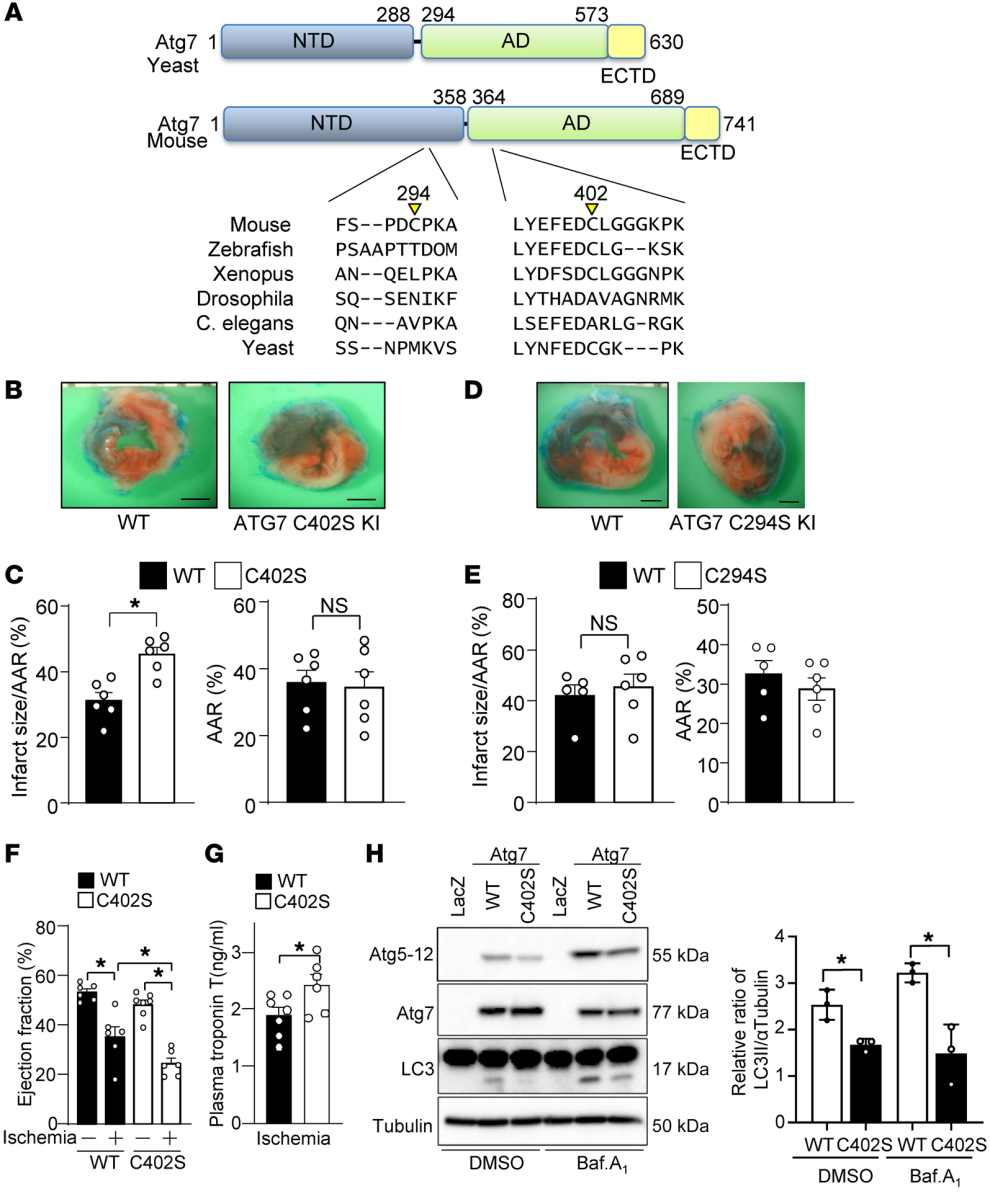
S-nitrosylation of Atg7 at Cys402 promotes Atg7 function and cardioprotection against ischemia. (**A**) Schema showing the location of the Cys294 and Cys402 residues in Atg7. NTD, N-terminal domain; AD, adenylation domain; ECTD, extreme C-terminal domain. (**B**–**E**) Atg7 C402S, C294S, and WT mice were subjected to ischemia for 3 hours. Representative images of TTC staining are shown. Scale bars: 1 mm. The percentage area at risk (AAR) and myocardial infarct area/AAR (%) are shown. Error bars represent SEM. *n* = 5–6. (**F**) Echocardiographic measurements after 3 hours of ischemia. *n* = 5–7. (**G**) Plasma troponin T levels were evaluated after 3 hours of ischemia. *n* = 6. (**H**) Atg7-KO mouse embryonic fibroblasts were transduced with Ad-LacZ, Ad-Atg7 WT, or Ad-Atg7 C402S for 24 hours. They were then subjected to bafilomycin A1 treatment for 2 hours. Protein samples were prepared and Western blotting was performed to detect LC3, Atg7, Atg12, and α-tubulin. *n* = 3. *P < 0.05 by 2-tailed Student’s *t* test (**C**, **E**, **G**, and **H**) or 1-way ANOVA (**F**).

Atg7 Cys294 is located in the N-terminal domain (NTD), whereas Cys402 is located in the adenylation domain, which is important for Atg7 binding to Atg8 (LC3) and MgATP (Figure 7A). Compared with Cys294, Cys402 is relatively well conserved among species. In order to evaluate the functional significance of Atg7 S-nitrosylation in vivo, we generated Atg7-C294S–KI and Atg7-C402S–KI mice on the C57BL/6J background. None of the heterozygous mice from either line exhibited a significant phenotype in the heart or any other organ at baseline. When these mice were subjected to 3 hours of ischemia, Atg7-C402S–KI mice exhibited larger infarcts than WT mice (Figure 7, B and C). However, Atg7-C294S–KI mice did not show significant enlargement of infarct size compared to WT mice (Figure 7, D and E). Atg7-C402S–KI mice also exhibited decreases in ejection fraction and increases in plasma troponin T levels (Figure 7, F and G, and Supplemental Table 3). These results suggest that S-nitrosylation of Atg7 at Cys402 plays a significant role in inhibiting myocardial injury during myocardial ischemia. We expressed either WT Atg7 or Atg7 C402S in Atg7-deficient mouse embryonic fibroblasts and evaluated the impact of S-nitrosylation of Atg7 on autophagy by monitoring the capacity of Atg7 to bind to either Atg5 or LC3. As shown in Figure 7H, WT Atg7 promotes formation of the Atg5-Atg12 complex, which was inhibited with Atg7 C402S. In addition, WT Atg7 promoted LC3-I to LC3-II conversion, which was significantly attenuated with Atg7 C402S. Taken together, these results suggest that S-nitrosylation of Atg7 at Cys402 positively regulates the E1-like activity of Atg7 and activation of autophagy.

## Discussion

**Figure 8:**
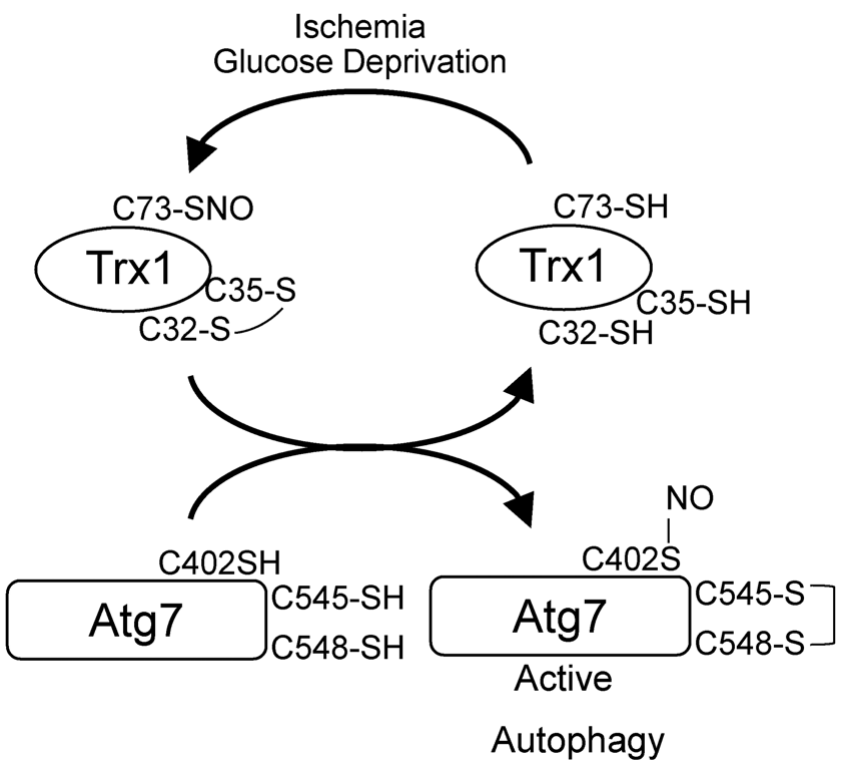
Schematic representation of Trx1-mediated Atg7 regulation. Trx1 is oxidized at Cys32-Cys35 in response to GD, which is prerequisite for S-nitrosylation at Cys73. Cys32-Cys35 are reduced by Cys545-Cys548 of Atg7, which triggers S-nitrosylation of Atg7 at Cys402. S-nitrosylation of Atg7 promotes autophagy.

We here show that Trx1 promotes autophagy during myocardial ischemia through transnitrosylation of Atg7. Although Trx1 is oxidized during stress, oxidation of its catalytic domain allows S-nitrosylation at Cys73. This NO is transferred to Atg7 at Cys402 when thiol-disulfide exchange takes place between the CXXC motifs on Atg7 (at Cys545-Cys548) and Trx1 (at Cys32-Cys35), which in turn promotes autophagy (Figure 8). Our results suggest that the thiol-disulfide exchange and transnitrosylation are functionally coupled, allowing Trx1 to stimulate cell protective mechanisms even when Trx1 is oxidized at Cys32-Cys35 in its oxidoreductase catalytic center in the presence of an oxidative environment.

### Trx1 acts as a transnitrosylase in cardiomyocytes during ischemia

S-nitrosylation of proteins takes place when proteins are directly exposed to NO produced by NO synthase (NOS) or small NO carrier molecules, such as N_2_O_3_ and GSNO. However, protein S-nitrosylation takes place more efficiently and in a more stable manner when NO is transferred from other NO-carrying proteins, including Trx1 (20–24), GAPDH (15), and hemoglobin (34). This process is called transnitrosylation (14). Transnitrosylation is a secure and regulated mechanism of amplification of the NO/SNO signal since it can target protein thiols through protein-protein interaction (33). Here we show that Trx1 acts as an endogenous transnitrosylase for cardiac proteins, including Atg7, under basal conditions and during myocardial ischemia, and that Cys73 plays an essential role in transferring NO to its targets. The ability of Trx1 to transnitrosylate proteins has been shown primarily in vitro. However, we present multiple lines of evidence that the transfer of NO from Cys73 of Trx1 to Atg7 occurs in the heart in vivo and that transnitrosylation is critical for activation of autophagy. Trx1-mediated transnitrosylation may be particularly important during hypoxia/ischemia because (although it is controversial; see ref. 35), in theory, the level of free NO may be reduced in this condition since O_2_ is required for NO synthesis (36, 37). Furthermore, NO is highly reactive but has limited bioavailability (15). Recent reports indicated that S-nitrosylation of cellular proteins involves a series of enzymatic reactions and signaling within an interactome that mediates NO synthesis, SNO synthesis, and transnitrosylation (33, 38). Our findings are consistent with these findings in that Trx1 and Atg7 form at least a transient protein complex after ischemia. The presence of a Trx1 interactome to facilitate S-nitrosylation would increase the specificity, stability, and significance of the transnitrosylation of Atg7 by Trx1.

#### The molecular mechanisms of transnitrosylation by Trx1.

Trx1 is an S-nitrosylated protein that can either receive NO from or transfer NO to other proteins (11). The transnitrosylase activity of Trx1 is associated with S-nitrosylation at Cys73 (23). S-nitrosylation of Trx1 at Cys73 is stimulated when the oxidoreductase catalytic site, namely Cys32-Cys35, is oxidized. Thus, it is likely that SNO is released from Cys73 of Trx1 when Trx1 is reduced at Cys32-Cys35 (32). Interestingly, Atg7 has a Trx1-like CXXC motif at Cys545-Cys548 that can reduce Trx1 through thiol-disulfide exchange. Furthermore, the transfer of NO from Trx1 to Atg7 is significantly inhibited when Atg7 CC545/548SS, which inhibits the thiol-disulfide exchange between Atg7 and Trx1, is expressed. Thus, the thiol-disulfide exchange not only allows Trx1 to release SNO from Cys73, possibly through an allosteric effect of the reduction of the catalytic center, but also facilitates the transfer of NO from Trx1 to Atg7 due to their proximity. The results of an unbiased screening suggest that many proteins whose S-nitrosylation is dependent on the presence of intact Cys73 in Trx1 have CXXC-like motifs, and, by inference, oxidoreductase-like activity. In addition to Atg7, we found that Fhl2 directly reduces Trx1 in vitro (Figure 5H). RhoA, an S-nitrosylation target of Trx1 Cys73 (39), also possesses a CXXC-like motif. Thus, thiol-disulfide exchange may also facilitate the transfer of NO from Trx1 at Cys73 to other proteins.

#### Pathophysiological significance of Trx1-induced protein oxidation.

Trx1 is generally thought of as a reducing enzyme. However, our results show that Trx1 actually oxidizes Atg7, whereas Atg7 reduces Trx1 under GD conditions. Of note, the substrate-trapping Trx1 mutant can interact with Atg7 at Cys545-Cys548. It is generally thought that Cys32 of the trapping mutant is reduced at baseline and then forms a disulfide bond with an oxidized substrate (40). However, given that Atg7 Cys545-Cys548 reduces the disulfide bond at Cys32-Cys35 of Trx1, it would appear that oxidized Cys32 may also form a disulfide with reduced substrates under some conditions. It should be noted that Trx1 mediates S-nitrosylation of Atg7 even under basal conditions, without GD or ischemia, in the heart in vivo (Figure 5C). This is consistent with the fact that approximately half of Trx1 remains oxidized in the heart under basal conditions (23). The ability of Trx1 to protect the heart through transnitrosylation even after it loses its oxidoreductase activity suggests that the previously reported salutary actions of Trx1 may be mediated through multiple mechanisms besides the antioxidant mechanism.

#### S-nitrosylation of Atg7 positively regulates autophagy.

The role of S-nitrosylation in the regulation of autophagy is not fully understood (reviewed in ref. 41). Atg7 is an E1 enzyme that binds to ubiquitin-like proteins Atg8 and Atg12 and thus plays an important role in the regulation of autophagy. Recent studies have reported that oxidation of catalytic thiols of Atg7 prevents LC3 lipidation and impairs autophagy (31). Under normal conditions, Atg7 is bound to LC3, and this prevents oxidation of Atg7. However, when LC3 is lipidated to phosphatidylethanolamine, the thiols in Atg7 become available for oxidation, negatively regulating autophagy. Our results suggest that S-nitrosylation of Atg7 promotes its activity and, hence, increases autophagy in the heart during stress conditions. S-nitrosylation of Atg7 may protect it from irreversible oxidation such as sulfonic acid formation. In addition, Trx1-induced disulfide bond formation in Atg7 may also protect it from irreversible oxidation since the disulfide bond is reversible. It is possible that Trx1 interacts with Atg7 to maintain reversible oxidative modifications such as S-nitrosylation and the disulfide bond so that Atg7 remains available for binding to LC3 and Atg5 when these oxidized thiols are reduced. This suggests that Trx1 could be a key regulator of Atg7 activity since it induces both S-nitrosylation and disulfide bond formation. We currently do not know why Atg7-C402S–KI mice exhibited more significantly enhanced myocardial infarction than Atg7-C294S–KI mice in response to myocardial ischemia. Further experiments are needed to address this issue.

Although the current study demonstrates the importance of the transnitrosylation activity of Trx1 during myocardial ischemia, the cellular level of S-nitrosylation is also regulated by enzymes that promote protein denitrosylation, including GSNO reductase (GSNOR) (42). GSNOR regulates intracellular levels of GSNO, an important bioavailable reserve of NO in cells. Mice lacking GSNOR exhibited protection against myocardial injury (43, 44). Thus, it is important to understand how the balance between transnitrosylation and denitrosylation is controlled in the stressed heart. Interestingly, it has been shown that Trx1 and Trx1 reductase negatively affect GSNO (45). Thus, it will be interesting to elucidate the interaction between the Trx1 system and the GSNO system.

We show that thiol-disulfide exchange between Trx1 and Atg7 and S-nitrosylation of Trx1 and Atg7 take place in cultured cardiomyocytes in vitro. We also show that S-nitrosylation of Atg7 during ischemia was attenuated in cardiac-specific Trx1-KO mice. Thus, our results indicate that Trx1-mediated transnitrosylation of Atg7 is important in cardiomyocytes. Since Trx1-C73S–KI and Atg7-C402S–KI mice are systemic KI mice, however, we cannot exclude involvement of Trx1-mediated Atg7 transnitrosylation in non-myocytes in mediating the protection against myocardial ischemia. We found that, as in cardiomyocytes, GD-induced S-nitrosylation of Trx1 also takes place in cardiac fibroblasts. Thus, the functional significance of Trx1-mediated Atg7 transnitrosylation in various cell populations in the heart remains to be further clarified using unbiased approaches, including single-cell RNA sequencing analyses.

We speculate that Atg7-mediated activation of autophagy protects the heart against ischemia through preservation of ATP and the cellular quality-control mechanisms. However, contributions of other mechanisms cannot be excluded and further investigation is warranted. For example, ATAC sequencing analyses may clarify the chromatin accessibility and transcriptome influenced by the Trx1-Atg7–induced protection against ischemia.

Although Trx1-C73S–KI mice exhibited larger myocardial injuries after 3 hours of ischemia than WT mice, their left ventricular function was maintained after 48 hours of fasting even though autophagic flux was inhibited. We speculate that the level of stress is greater in myocardial ischemia caused by permanent occlusion than during fasting. Thus, Trx1-mediated transnitrosylation of Atg7 is more important for protection against prolonged ischemia than against fasting.

Taken together, our results suggest that Trx1 promotes autophagy through transnitrosylation of Atg7, thereby protecting the heart against ischemia. Although myocardial ischemia primarily promotes both oxidation and S-nitrosylation of Trx1, concurrent reduction of Trx1 by Atg7 induces transnitrosylation of Atg7 at Cys402 and stimulation of the E1-like activity of Atg7. Thus, the oxidoreductase activity of Atg7 not only regenerates Trx1 but also stimulates transnitrosylation of Atg7 itself, thereby playing an essential role in mediating protection of the heart against myocardial ischemia.

## Methods

### Cardiomyocyte cultures.

Primary cultures of ventricular cardiomyocytes were prepared from 1-day-old Crl: (WI) BR Wistar rats (Charles River Laboratories). The left ventricle (LV) was isolated, and the tissue was digested with collagenase type IV (Sigma-Aldrich), 0.1% trypsin (Life Technologies), and 15 μg/mL DNase I (Sigma-Aldrich) to obtain a single-cell suspension. Cardiomyocyte-rich and non–myocyte-rich fractions were obtained by centrifugation through a discontinuous Percoll gradient. Cardiomyocytes were cultured in Dulbecco’s modified Eagle’s medium (DMEM)/F-12 medium supplemented with 5% horse serum, 4 μg/mL transferrin, 0.7 ng/mL sodium selenite (Life Technologies), 2 g/L bovine serum albumin (fraction V), 3 mM pyruvic acid, 15 mM HEPES, 100 μM ascorbic acid, 100 μg/mL ampicillin, 5 μg/mL linoleic acid, and 100 μM 5-bromo-2′-deoxyuridine (Sigma-Aldrich). Highly enriched cultures of non-myocytes in which cardiac fibroblasts were the major cell type were cultured with the same culture medium as above except that 5-bromo-2′-deoxyuridine was not used. The medium was changed to a serum-free medium for 24 hours before any experiment. Isolation and culture of AMCMs were conducted according to a protocol described previously (46). This method utilizes direct needle perfusion of the LV ex vivo, which allows isolation, separation, and culture of AMCMs.

### Cell lines.

HEK293 cells (ATCC, CRL-1573) cultured in DMEM supplemented with 5% fetal bovine serum (FBS) were used for preparing adenoviruses. *Atg7*-null mouse embryonic fibroblast cells (courtesy of Mondira Kundu, St. Jude Children’s Research Hospital, Memphis, Tennessee, USA, and Maasaki Komatsu, Juntendo University, Tokyo, Japan) were cultured in DMEM with 5% FBS and used for evaluating autophagy and Atg7 activity assays.

### Generation of adenoviruses.

Briefly, pBHGloxΔE1,3 Cre (Microbix), including the ΔE1 adenoviral genome, was cotransfected with the pDC316 shuttle vector containing the gene of interest into HEK293 cells using Lipofectamine 2000 (Invitrogen). By homologous recombination, the gene of interest integrated with the E1-deleted adenoviral genome. The adenovirus thus generated was propagated in HEK293 cells as previously described (47). Adenoviruses harboring human WT Trx1 (Ad Trx1-WT), Trx1 C35S (Ad Trx1 C35S), Trx1(C73S) [Ad Trx1(C73S)], and human WT Atg7 (Ad Atg7-WT), Ad-Tf-LC3, and Ad-LacZ (control) have been described previously (2, 48, 49). Atg7 mutants in the pDC316 backbone vector (Atg7 C294S, Atg7 C402S, Atg7 CC294/402SS, Atg7 C545S, Atg7 C548S, and Atg7 CC545/548SS) were generated using the Quik-Change Mutagenesis Kit (Agilent) and adenoviruses were generated with the AdMax system (Microbix) with the shuttle vector pDC316-Atg7 and the mutant vectors. For knockdown of a protein, adenoviral transduction was carried out for 96 hours, whereas overexpression was achieved by transduction for 48 hours.

### Biotin switch assays.

Cardiomyocytes were harvested in Lysis Buffer (Cayman Chemicals) and then adjusted for equal protein concentration using the BCA assay (Thermo Fisher Scientific).

Intact mouse hearts were harvested at the mentioned time points after ischemia. The heart samples were kept on ice in the dark until all samples were harvested. The ischemic portion of the heart was used to prepare protein samples. The intact heart tissue was kept in the dark after harvest, and all procedures, including protein homogenization, protein concentration estimation, and biotin switch assays, were conducted in the dark room. After normalization of protein concentration using the BCA assay, the samples were subjected to a modified biotin switch assay (S-Nitrosylated Protein Detection Kit, Cayman Chemicals). Only fresh heart tissue was used for biotin switch assays. To detect a specific S-nitrosylated protein, S-nitrosylated proteins were enriched using streptavidin-agarose beads (Thermo Fisher Scientific, 20347) and immunoblotting using the antibody of interest was carried out.

### SDS-PAGE and Western blotting.

Heart homogenates and cardiomyocyte lysates for SDS-PAGE and immunoblotting analyses were prepared in RIPA lysis buffer containing 50 mM Tris (pH 7.5), 150 mM NaCl, 0.1% SDS, 1% Triton X-100, 1% sodium deoxycholate, 1 mM EDTA, 1 mM sodium orthovanadate, 1 mM sodium fluoride, and 1× Halt Protease inhibitor cocktail (Thermo Fisher Scientific). After determining the protein concentrations by BCA assay, equal amounts of protein were loaded on an SDS-PAGE gel with 4× Laemmli sample buffer (200 mM Tris-HCl pH 6.8, 40% glycerol, 8% SDS, 0.4% bromophenol blue, 10% β-mercaptoethanol). For separation under nonreducing conditions, β-mercaptoethanol was not added to the loading buffer. Proteins were then transferred to a PVDF membrane and immunoblotting was carried out using relevant antibodies. Densitometric analyses of the blots were carried out using the public domain ImageJ program (NIH). See complete unedited blots in the supplemental material.

### Antibodies.

Antibodies against the following proteins were used in this study: Trx1 (Cell Signaling Technology [CST], 2429), LC3 (MBL Intl. Corp., M186-3), Atg7 (CST, 8558), Atg5 (CST, 12994), Atg12 (CST, 4180), GAPDH (CST, 2118), α-cardiac actin (Novus, NBP2-61474), Fhl2 (Novus Biologicals, NBP1-31262), and α-tubulin (Sigma-Aldrich, T6199).

### Co-IP assays.

Cardiomyocyte lysates were prepared using lysis buffer (50 mM Tris-HCl pH 8.0, 150 mM sodium chloride, 1% IGEPAL). The homogenates were incubated on ice for 30 minutes and then centrifuged at 13,200*g* for 10 minutes at 4°C. The supernatants were then incubated with anti-FLAG–agarose beads (Sigma-Aldrich) for 4 hours at 4°C. After IP, the samples were washed 3–4 times and the bound proteins were then eluted using 2× SDS sample buffer (120 mM Tris-HCl pH 6.8, 20% glycerol, 4% SDS, 0.02% bromophenol blue, 10% β-mercaptoethanol).

### TMT analysis.

To identify the S-nitrosylation of proteins and their relative quantitation in cardiac-specific Trx1-KO and WT mice, we combined biotin switch and TMT labeling in one experiment. In brief, the same amount of protein extract from each WT or Trx1-KO mouse heart (*n* = 3) was biotin switched to convert the S-nitrosylation on cysteine into the more stable biotin-HPDP modification. After a protein assay using the BCA method, 100 μg of protein from each sample was in-solution digested without reduction and alkylation. The resulting peptides were labeled with 6-plex TMT reagents. After the labeling, 6 samples were mixed and desalted using a C18 cartridge, followed by high-pH RPLC separation. Ten percent of the samples was used for global protein expression quantitation and the rest was used for affinity enrichment of HPDP-modified peptides to study transnitrosylation. For global protein expression, a total of 16 fractions were collected and further analyzed by RPLC-MS/MS on a Q Exactive Hybrid Quadrupole-Orbitrap Mass Spectrometer (Thermo Scientific). The MS/MS spectra were searched against the SwissProt mouse protein database using the MASCOT (v.2.3) search engine on the Proteome Discoverer (v1.4) platform (https://www.matrixscience.com/server.html). In order to evaluate the level of S-nitrosylation, avidin enrichment was performed to enrich HPDP-modified peptides. The peptide quantitation was determined by TMT labeling. The sample WT-1 was used as a reference to determine relative changes in the identified proteins in other samples. Changes in global proteins and SNO-peptides that passed the *t* test (*P* ≤ 0.05) with ratios of 1.2-fold or higher or 0.8-fold or lower were considered significant.

### BIAM pull down.

Cardiomyocytes were lysed with lysis buffer (50 mM Tris pH 7, 150 mM NaCl, 0.5% NP-40, Protease Inhibitor Cocktail [Sigma-Aldrich]) containing 200 μM BIAM. Biotin-labeled proteins were pulled down with Avidin resin (Promega, V201A). The samples were eluted using 2× SDS sample buffer (120 mM Tris-HCl pH 6.8, 20% glycerol, 4% SDS, 0.02% bromophenol blue, 10% β-mercaptoethanol).

The peptides from both in-solution and in-gel trypsin digestion were desalted with C18 cartridges prior to LC-MS/MS analysis. The analysis was performed on either a Q Exactive mass spectrometer or a Fusion Lumos tribrid mass spectrometer coupled with U3000 nano LC system (Thermo Scientific). The peptides were separated using a C18 reversed-phase nano column (Acclaim PepMap RSLC, 75 μm × 50 cm, 2 μm, 100 Å, Thermo Fisher Scientific). The eluted peptides were directly introduced into the MS system with a spray voltage of 2 kV and a capillary temperature of 275°C. All the spectra were acquired in positive mode with data-dependent analysis. The precursor was selected and fragmented using higher energy collision dissociation (HCD) fragmentation at collision energy set at 30%. The MS/MS spectra were searched against a Uniprot database with corresponding species using either a local MASCOT search engine (v.2.3) or a Sequest search engine on the Proteome Discoverer platform. Proteins and peptides were identified with a false discovery rate (FDR) of less than 1%. For TMT analysis, the peptides were further filtered using Scaffold software (https://www.proteomesoftware.com) with a 95% confidence interval. For disulfide bond mapping, SIM-XL software (http://patternlabforproteomics.org/sim-xl/) was used.

### Cell viability.

Cell viability was measured using CellTiter-Blue (Promega) according to the manufacturer’s instructions.

### Evaluation of autophagy.

A key step in autophagy is the conversion of LC3-I to LC3-II. Measurement of this conversion is the gold standard to evaluate autophagy. We studied this using (a) Western blot analysis of LC3 and (b) fluorescence tagging of LC3 using tandem-fluorescent mRFP-GFP-LC3 and GFP-LC3-RFP. The amount of LC3-II, measured by Western blot, is a direct indicator of the abundance of autophagosomes. In order to measure the extent of LC3-II delivery to lysosomes (i.e., to monitor autophagic flux), mRFP-GFP-LC3 was used. Because mRFP, but not GFP, is resistant to lysosomal acids, autophagosomes that fluoresce both red (mRFP) and green (GFP) are not yet fused with lysosomes, whereas those puncta detectable only in red are associated with autophagosomes fused with lysosomes (autolysosomes). In the GFP-LC3-RFP system, GFP-LC3-RFP is cleaved by Atg4B into an identical amount of GFP-LC3 and RFP. GFP-LC3 is degraded by autophagy, whereas RFP remains and serves as an internal control. When autophagy is activated, the GFP/RFP ratio is reduced. Computational images showing the GFP/RFP ratio were processed with NIS-Elements software (Nikon).

Autophagy was assessed by the evaluation of LC3 isoforms and counting of mRFP and GFP puncta as described previously (28, 29, 50, 51). LC3-II accumulation was evaluated by immunoblotting at baseline and after 4 hours of GD in the presence and absence of bafilomycin A1, which inhibits lysosomal activity. To evaluate autophagy, cardiomyocytes cultured on coverslips were transduced with Ad Tf-LC3 or Ad GFP-LC3-RFP. After 48 hours, cells were washed with PBS, fixed with 4% paraformaldehyde in PBS, and then mounted with a reagent containing DAPI (Vectashield, Vector Laboratories). Green and red puncta were counted using a fluorescence microscope and GFP/RFP ratio images were processed using NIS-Elements software. These procedures have been described previously (52).

### Measurement of Atg7 activity.

The activity of Atg7 was evaluated as described previously (26). *Atg7*-null mouse embryonic fibroblast cells were transduced with adenovirus harboring *lacZ*, Atg7 WT, or Atg7 CC298/406SS mutant for 24 hours and were treated with bafilomycin A1 for 2 hours. Protein lysates were prepared as described above. Conversion of LC3-I to LC3-II and the levels of free Atg5 in comparison with conjugated Atg5-Atg12 were measured by Western blotting. To obtain a direct measure of Atg7 activity, Atg7 bound to GFP-LC3 was measured. *Atg7*-null mouse embryonic fibroblast cells were transduced with *lacZ*, Atg7 WT, or Atg7 C402S mutant along with GFP-LC3 for 24 hours. Protein samples were subjected to pull-down using a GFP-trap kit to detect proteins bound to GFP-LC3 according to the manufacturer’s instructions (Chromtek).

### Mouse models.

Trx1-C73S was generated by mutating Cys73 to Ser73 using site-directed mutagenesis of human Trx1 cDNA. Transgenic mice with cardiac-specific expression of Tg-Trx1(C73S) were generated on the FVB background, using the *Myh6* promoter (courtesy of J. Robbins, University of Cincinnati, Cincinnati, Ohio, USA). Cardiac-specific Trx1-KO knockout mice were described previously (53). Trx1-C73S–KI mice were generated on the C57BL/6J background. The targeting vector was generated by PCR using BAC clones RP23-37K11 and RP23-347N15 from the C57BL/6J library as template. The C73S mutation was introduced by mutating TGC to TCC in exon 4 of the *Trx1* gene. The targeting vector included a neomycin cassette for positive selection and diphtheria toxin A (DTA) for negative selection. The neomycin cassette was self-deleted during germline transmission. ES cells containing the mutation were screened by Southern blotting and then propagated in WT mice (Cyagen). KI of the Trx1 gene was confirmed by genotyping and sequencing. Atg7-C294S–KI mice and Atg7-C402S–KI mice were generated using CRISPR/Cas9 technology to create point mutations. These mice were also generated on the C57BL/6J background. KI of the *Atg7* gene was confirmed by genotyping and sequencing. The presence in both alleles was ensured by cloning and sequencing of the plasmid. All experiments were conducted on 3- to 5-month-old mice. Trx1-C73S–KI mice used in this study were homozygous, whereas cardiac-specific Trx1-KO and Atg7-KI mice were heterozygous.

### Echocardiography.

Mice were anesthetized using 12 μL/g body weight of 2.5% Avertin (Sigma-Aldrich), and echocardiography was performed using ultrasonography (Acuson Sequoia C256; Siemens Medical Solutions). A 13-MHz linear ultrasound transducer was used.

### Ischemia surgery in mice.

The method used to apply myocardial ischemia in mice has been described previously (48, 54, 55). Mice were housed in a temperature-controlled environment with 12-hour light/dark cycles where they received food and water ad libitum. Mice were anesthetized by intraperitoneal injection of 60 mg/kg of pentobarbital sodium. A rodent ventilator (Model 683, Harvard Apparatus Inc.) was used with 65% oxygen during the surgical procedure. The animals were kept warm using heat lamps and heating pads. Rectal temperature was monitored and maintained between 36.8°C and 37.2°C. The chest was opened by a horizontal incision through the muscle between the ribs via the third intercostal space. The anterior descending branch of the left coronary artery (LAD) was ligated using an 8-0 nylon suture with silicon tubing (1 mm OD) placed on top of the LAD, 2 mm below the border between the left atrium and LV, to achieve ischemia. Regional ischemia was confirmed by ECG change (ST elevation). Prolonged ischemia was carried out for 3 hours.

### Assessment of area at risk and infarct size by TTC staining.

After prolonged ischemia for 3 hours, mice were anesthetized again and intubated. The heart was arrested at the diastolic phase by KCl injection. The ascending aorta was cannulated and perfused with saline to wash out the blood. Following LAD occlusion, Alcian blue dye (1%) was perfused into the aorta and coronary arteries. The heart was then excised. The LV was sliced into six 1-mm-thick cross sections. The LV sections were then incubated in a 1% triphenyltetrazolium chloride solution (TTC) at 37°C for 10 to 12 minutes, followed by incubation in 10% formaldehyde for 2 hours. Images of both sides of the LV section were captured, the infarct area (white), area at risk (AAR, not blue), and the total LV area were measured using ImageJ, and the values were averaged. The percentages of infarct and AAR in each section were multiplied by the weight of that section and then totaled from all sections. AAR/LV and infarct size/AAR are expressed as percentages.

### Measurement of plasma troponin T.

Blood was collected and allowed to coagulate at room temperature for 10 minutes, followed by centrifugation at 375*g* for 15 minutes. The clear supernatant was collected and assayed for troponin T levels using an ELISA kit following the manufacturer’s protocol (AFG Biosciences, EK731778). Briefly, 10 μL of each sample was diluted in 40 μL sample dilution buffer (1:5 dilution) and loaded into the anti–troponin T antibody–coated ELISA plate well. After incubation at 37°C for 30 minutes, wells were washed with wash buffers and incubated with HRP-conjugated secondary antibody. After incubation with HRP-conjugated antibody and following buffer washes, the chromogen substrates A and B were added, turning the samples blue. The reaction was stopped by adding stop solution, turning the color yellow. The absorbance was read at 450 nm within 15 minutes of stopping the reaction. The optical density was plotted, and the concentration of each sample was calculated, including the dilution factor, by comparison with the optical density of a known standard concentration of troponin T provided in the kit.

### In vitro redox reaction.

Recombinant Atg7 (Novus Biologicals, E-318) or His-tagged Fhl2 (Novus Biologicals, NBP2-51868) (4–7.2 μg) was incubated with a reaction buffer (100 mM Tris pH 6.8, 150 mM NaCl) with 1 mM DTT for 1 hour. DTT was removed from Atg7 by dialysis with a centrifugal filter (Amicon Ultra-0.5, UFC500396). For His-tagged Fhl2, which could not be recovered from the centrifugal filter, Ni-NTA resin (Thermo Fisher Scientific, 88221) was used to remove DTT. To evaluate the redox reaction between Trx1 and Atg7, recombinant Trx1 (2–12 μg) was incubated with the Atg7 for 3 hours. Reduced thiols were labeled with 4 mM BIAM for 10 minutes for Western blot analysis or 50 mM *N*-ethylmaleimide (NEM) for 18 hours for MS analysis. For the redox reaction between Trx1 and Fhl2, 5 μg of recombinant Fhl2 was incubated with the reaction buffer (100 Tris pH 6.8, 150 mM NaCl) with 1 mM DTT and Ni-NTA resin for 1 hour. Fhl2 trapped on the resin was washed with 1 mL reaction buffer twice. Recombinant Trx1 (3 μg) was incubated with Fhl2 in the reaction buffer with 30 mM imidazole for 1 hour. Reduced thiols were labeled with 0.5 mM BIAM for 30 minutes. The labeling was terminated with sample buffer containing excess β-mercaptoethanol (320 mM final) and the sample was then analyzed by Western blotting.

### Statistics.

Data are expressed as mean ± SEM. Normality was tested with the Shapiro-Wilk normality test. If the data exhibited a normal distribution, pairwise testing was performed with a 2-tailed Student’s *t* test or multiple group comparisons were performed by 1-way ANOVA, followed by Tukey’s post hoc test. If the data failed normality testing, pairwise testing was performed with the nonparametric Mann-Whitney *U* test and multiple group comparisons were performed by the nonparametric Kruskal-Wallis test, followed by Dunn’s post hoc test. *P* less than 0.05 was considered statistically significant.

### Study approval.

All protocols concerning animal use were approved by the Institutional Animal Care and Use Committee of Rutgers New Jersey Medical School.

## Author contributions

NN and SO designed the study, performed most of the experiments, analyzed data, interpreted results, and wrote the manuscript. JN, RM, XX, SK, CH, EAS, WM, AST, KT, YM, and JF performed experiments and analyzed data. PZ performed surgical manipulations of mice. CW, TL, TC, and HL performed proteomic experiments, analyzed data, and interpreted results. JS designed and supervised the study, interpreted results, generated project resources, and wrote the manuscript. All authors reviewed and commented on the manuscript.

## Supplementary Material

Supplemental data

## Version 1 12/08/2022

In-Press Preview

## Version 2 02/01/2023

Electronic publication
