# Supplementary material for: Thioredoxin 1 promotes autophagy through transnitrosylation of Atg7 during myocardial ischemia
Source: J Clin Invest. 2023 Feb 1;133(3):e162326. doi: 10.1172/JCI162326 (PMC9888389; doi:10.1172/JCI162326)
Supplement: Supplemental data [file jci-133-162326-s091.pdf]

Data Supplement

Thioredoxin 1 promotes autophagy through transnitrosylation of Atg7 during myocardial ischemia

Narayani Nagarajan[1], Shin-ichi Oka[1], Jihoon Nah[1], Changgong Wu[2], Peiyong Zhai[1], Risa Mukai[1], Xiaoyong Xu[1,3], Sanchita Kashyap[1], Chun-Yang Huang[1,4,5], Eunah Sung[1], Wataru Mizushima[1], Allen Sam Titus[1], Koichiro Takayama[1], Youssef Mourad[1], Jamie Francisco[1], Tong Liu[2], Tong Chen[2], Hong Li[2], Junichi Sadoshima[1,6]

[1]Department of Cell Biology and Molecular Medicine, Cardiovascular Research Institute, Rutgers New Jersey Medical School, Newark, New Jersey, USA.

[2]Center for Advanced Proteomics Research, Department of Microbiology, Biochemistry, and Molecular Genetics, Rutgers New Jersey Medical School and Cancer Institute of New Jersey, Newark, New Jersey, USA.

[3]Department of Cardiology, Ningbo Medical Center Lihuili Hospital, Ningbo, Zhejiang, China.

[4]Division of Cardiovascular Surgery, Department of Surgery, Taipei Veterans General Hospital, Taipei, Taiwan.

[5]Institute of Clinical Medicine, School of Medicine National Yang-Ming University, Taipei, Taiwan.

NN and SO contributed equally to this study.

[6]Address correspondence to:
Junichi Sadoshima, MD, PhD
Department of Cell Biology and Molecular Medicine,
Cardiovascular Research Institute,
Rutgers New Jersey Medical School,
185 S. Orange Ave., MSB G609, Newark, NJ 07103, USA
Phone: +1-973-972-8916, FAX: +1-973-972-8919
E-mail: sadoshju@njms.rutgers.edu

Running title: **Trx1 transnitrosylates Atg7**

Figure S1

A

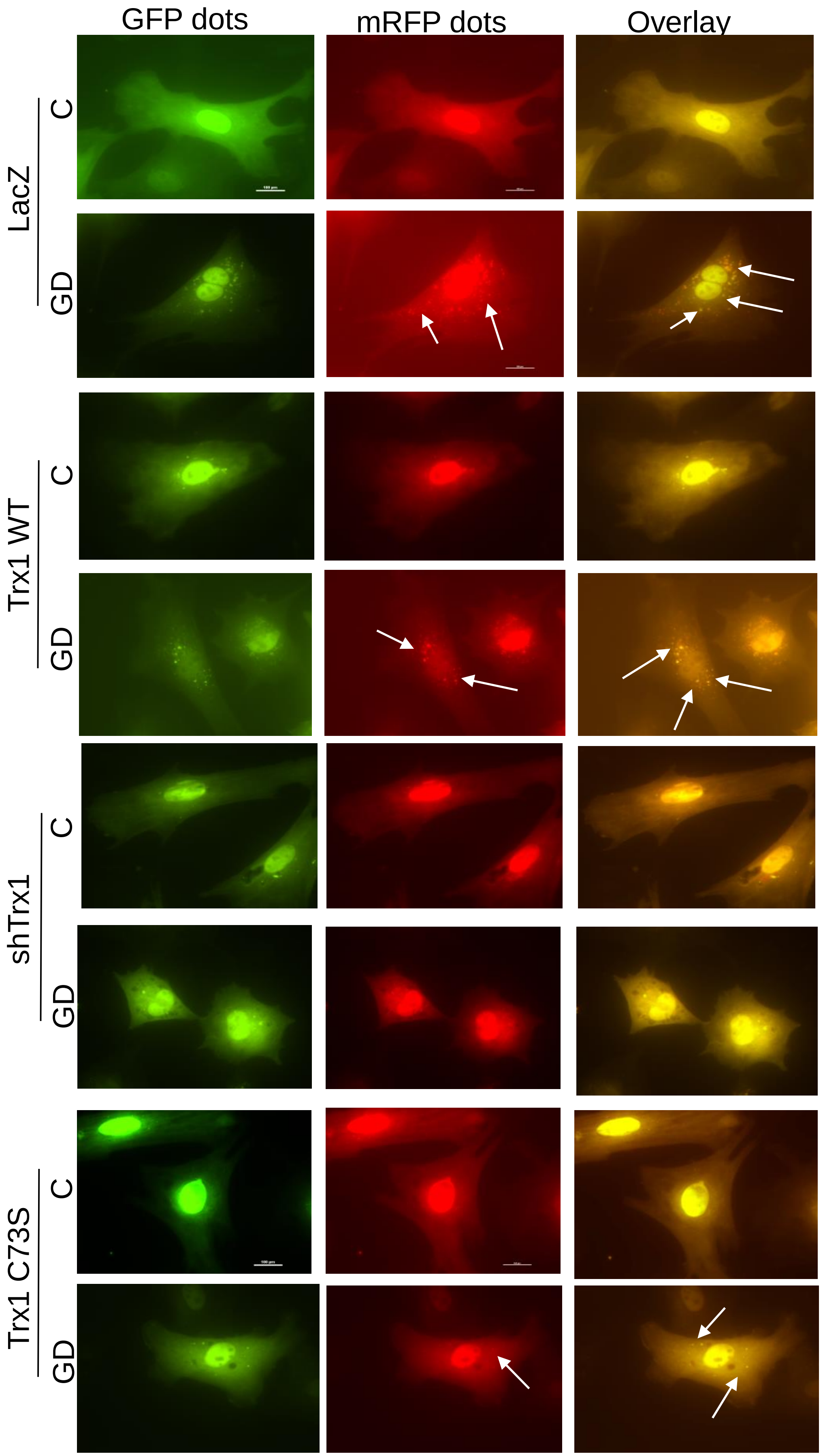

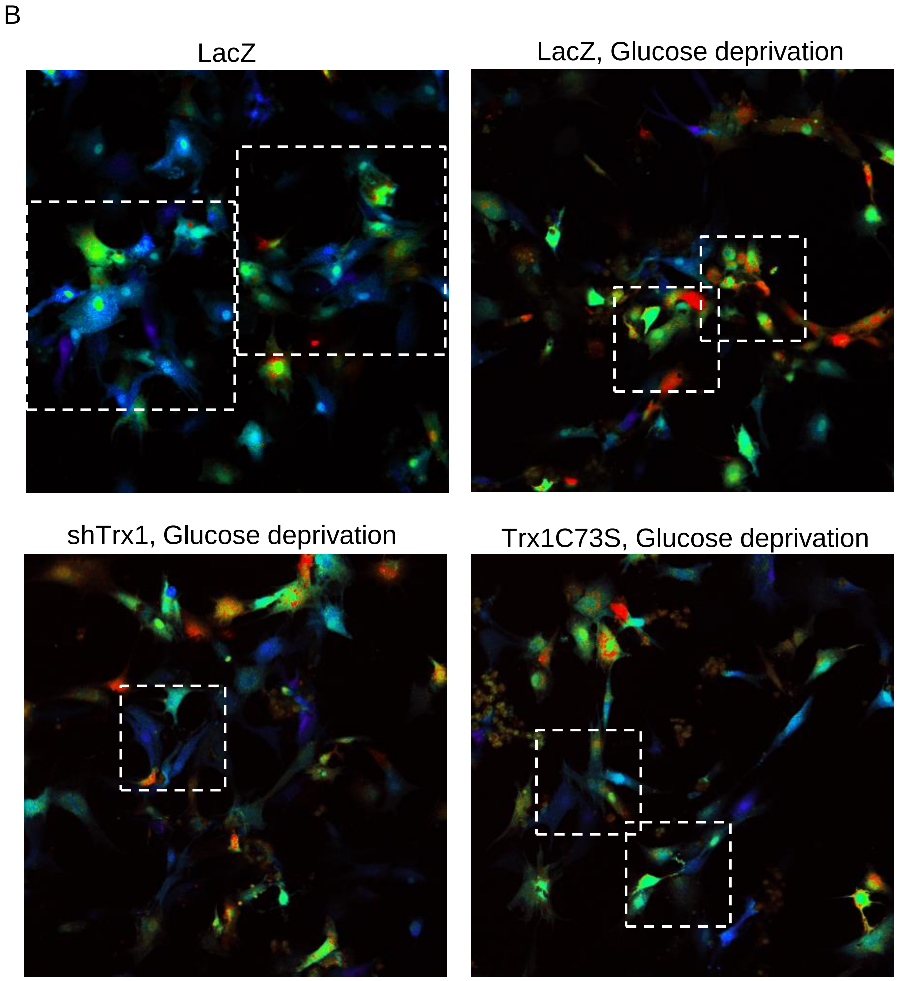
B
LacZ
LacZ, Glucose deprivation
shTrx1, Glucose deprivation
Trx1C73S, Glucose deprivation

Figure S1. (A) Representative microscopic images of GFP and mRFP dots shown in Figure 2A. (B) Low powered images corresponding to Figure 2B.

Table S1

Group	*n*	LVEDD	LVESD	IVSWT	PWT	EF (%)	%FS
**Wild**	**8**	4.19±0.28	3.07±0.38	0.73±0.06	0.87±0.12	52.49±9.86	26.95±6.06
**Wild Starvation**	**8**	3.94±0.59	2.93±0.63	0.71±0.09	0.69±0.11	51.83±9.37	26.35±5.78
**C73S**	**8**	4.14±0.22	3.11±0.32	0.65±0.05	0.61±0.09	49.35±9.2	24.39±5.63
**C73S Starvation**	**8**	3.8±0.71	2.82±0.43	0.69±0.16	0.67±0.24	49.25±11.8	24.89±7.38

Value are means ± SD. n, number; LVEDD, left ventricular end diastolic dimension, mm; LVESD, left ventricular end systolic dimension, mm; IVSWT, interventricular septal wall thickness, mm; PWT, posterior wall thickness, mm; EF, left ventricular ejection fraction, %; FS, fractional shortening, %. Statistical significance was determined with ANOVA. There is no statistical significance between Wild and Trx1C73S knock-in mice.

Table S2

Group	*n*	LVEDD	LVESD	IVSWT	PWT	EF (%)	%FS
**Wild**	**5**	4.46±0.27	3.22±0.24	0.56±0.07	0.51±0.07	54.30±2.31	27.97±1.41
**Wild MI**	**5**	4.76±0.50	3.96±0.67	0.43±0.05	0.45±0.09	35.93±10.54	17.38±5.59
**C73S**	**6**	4.45±0.29	3.4±0.32	0.63±0.09	0.54±0.05	47.33±4.89	23.65±2.86
**C73S MI**	**5**	4.72±0.26	4.23±0.25	0.53±0.05	0.41±0.05	22.59±5.71*	10.37±2.84*

Value are means ± SD. n, number; LVEDD, left ventricular end diastolic dimension, mm; LVESD, left ventricular end systolic dimension, mm; IVSWT, interventricular septal wall thickness, mm; PWT, posterior wall thickness, mm; EF, left ventricular ejection fraction, %; FS, fractional shortening, %. Statistical significance was determined with ANOVA. * $p<0.05$; vs Wild MI.

Table S3

Group	*n*	LVEDD	LVESD	IVSWT	PWT	EF (%)	%FS
**Wild**	**6**	4.58±0.35	3.33±0.33	0.61±0.07	0.52±0.09	53.27±3.35	27.38±2.03
**Wild MI**	**6**	4.65±0.34	3.87±0.49	0.51±0.09	0.47±0.05	35.17±10.09	16.94±5.33
**C402S**	**7**	4.37±0.40	3.32±0.35	0.58±0.08	0.49±0.05	48.21±4.86	24.15±2.84
**C402S MI**	**6**	4.31±0.44	3.82±0.37	0.47±0.08	0.41±0.10	24.47±5.21*	11.20±2.62*

Value are means ± SD. n, number; LVEDD, left ventricular end diastolic dimension, mm; LVESD, left ventricular end systolic dimension, mm; IVSWT, interventricular septal wall thickness, mm; PWT, posterior wall thickness, mm; EF, left ventricular ejection fraction, %; FS, fractional shortening, %. Statistical significance was determined with ANOVA. * $p<0.05$; vs Wild MI.

UNCUT GELS

Figure 1B

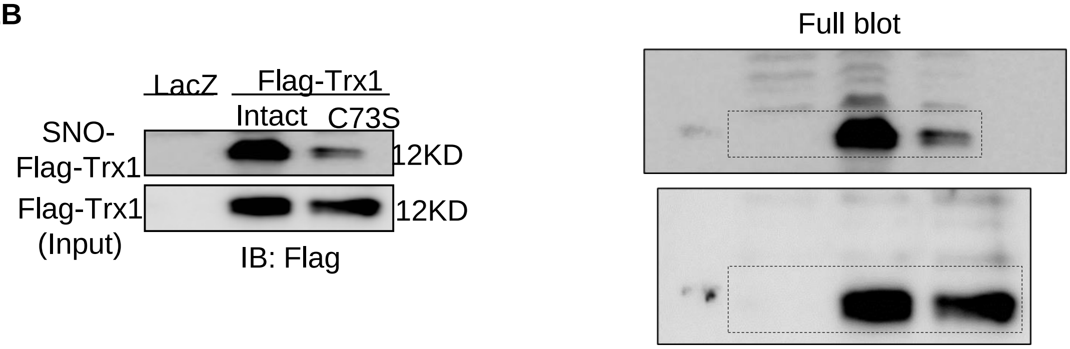

Figure 1D

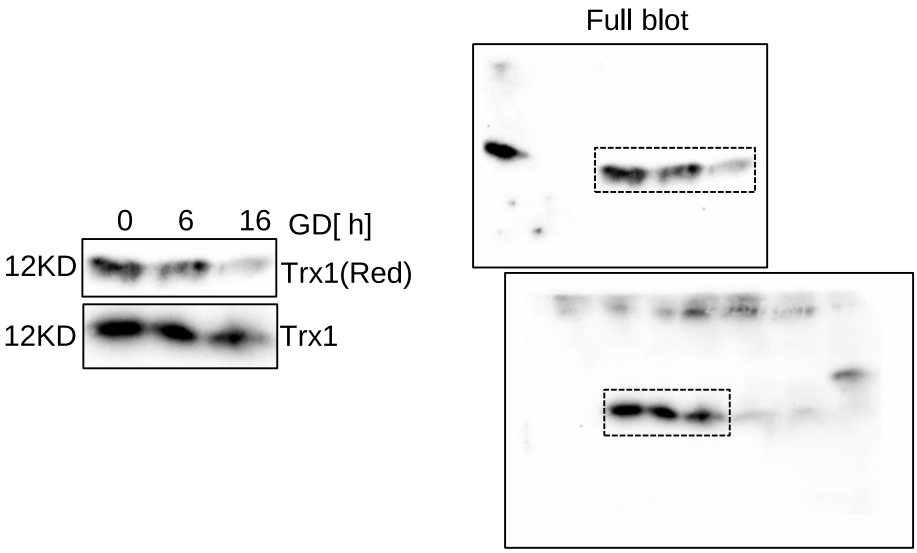

Figure 1E

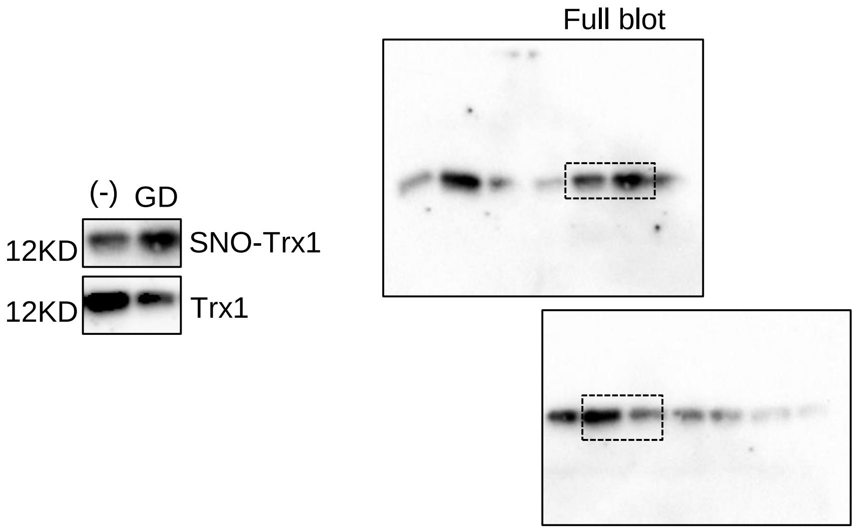

Figure 1F

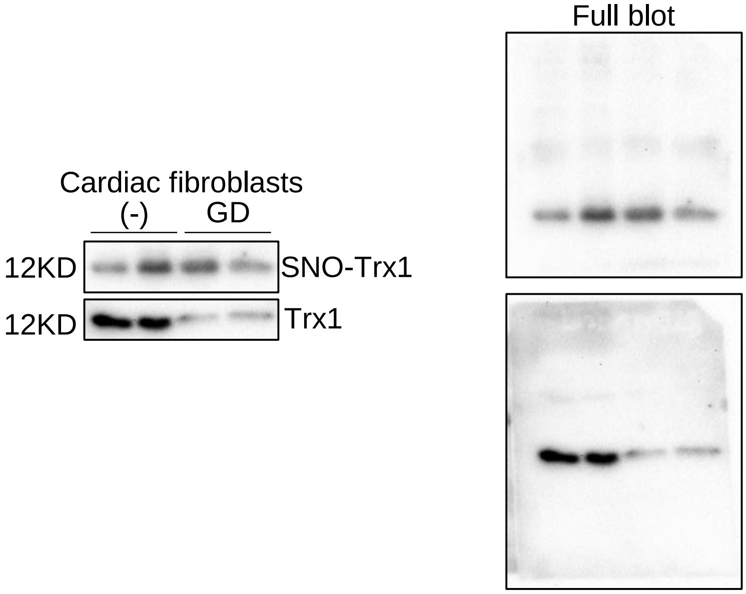

Figure 2D

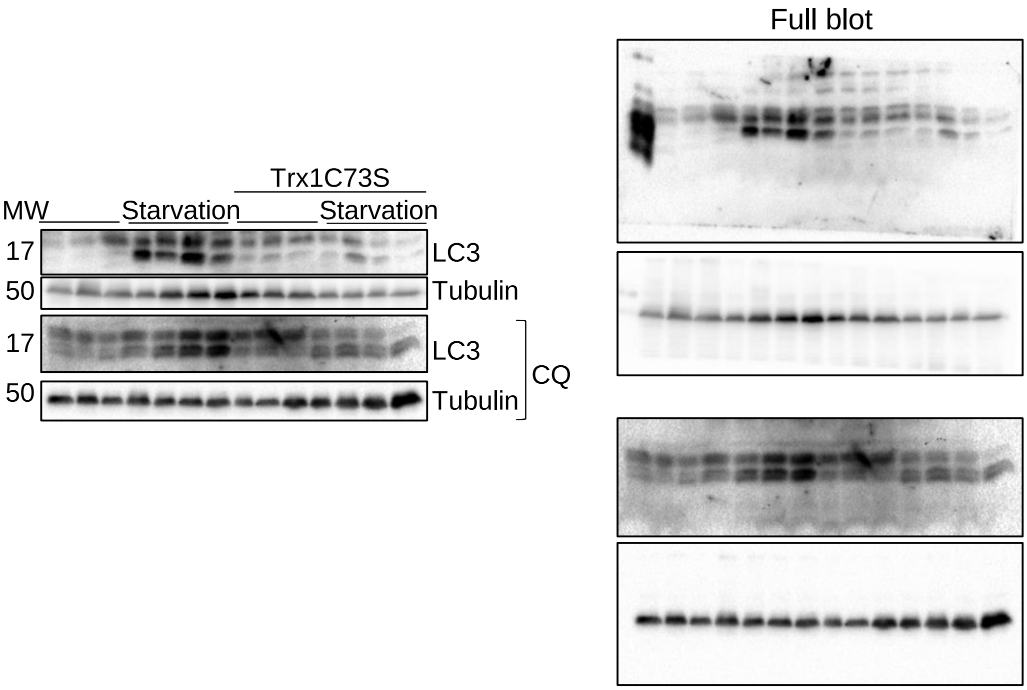

Figure 3A

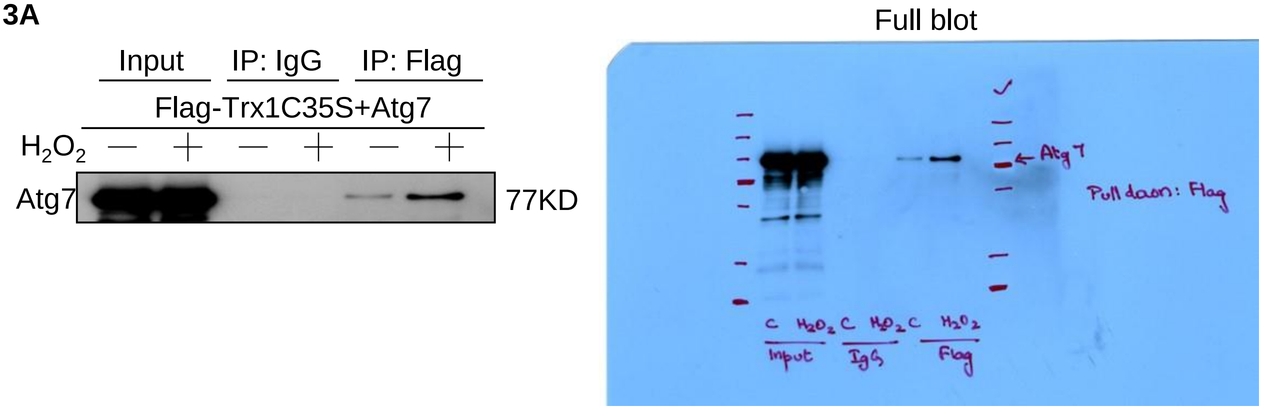

Figure 3B

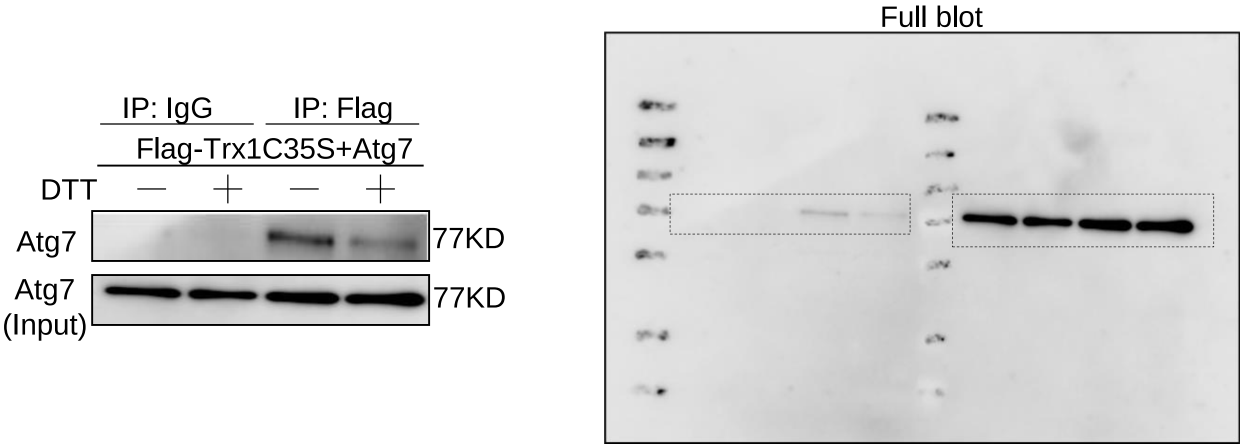

Figure 3C

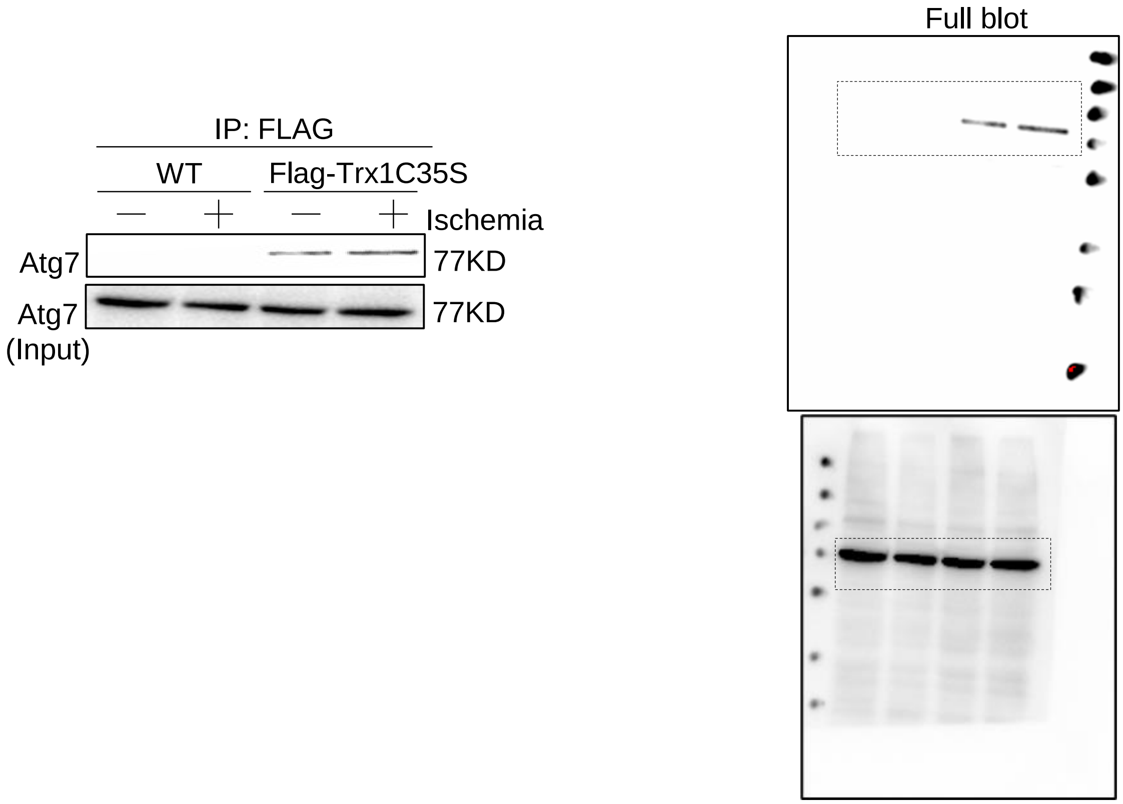

Figure 3D

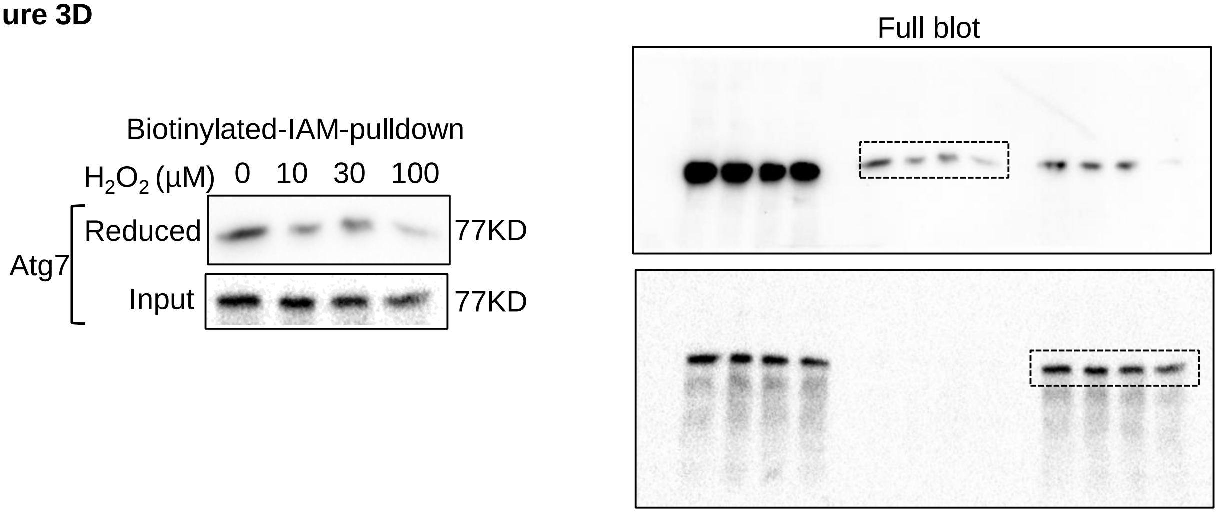

Figure 3F

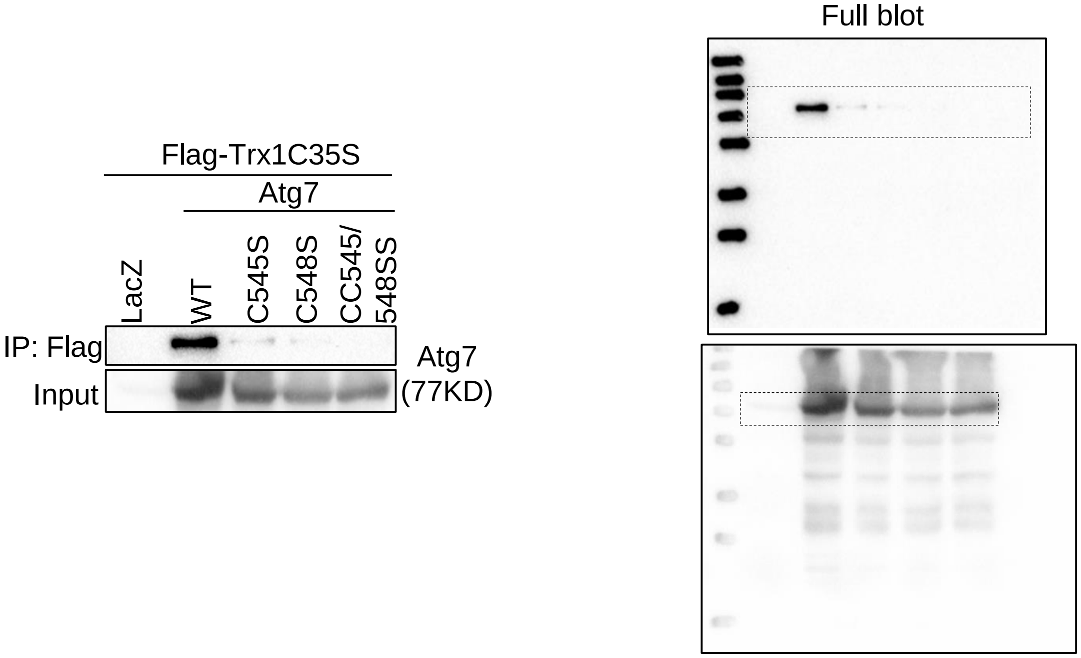

Figure 3G

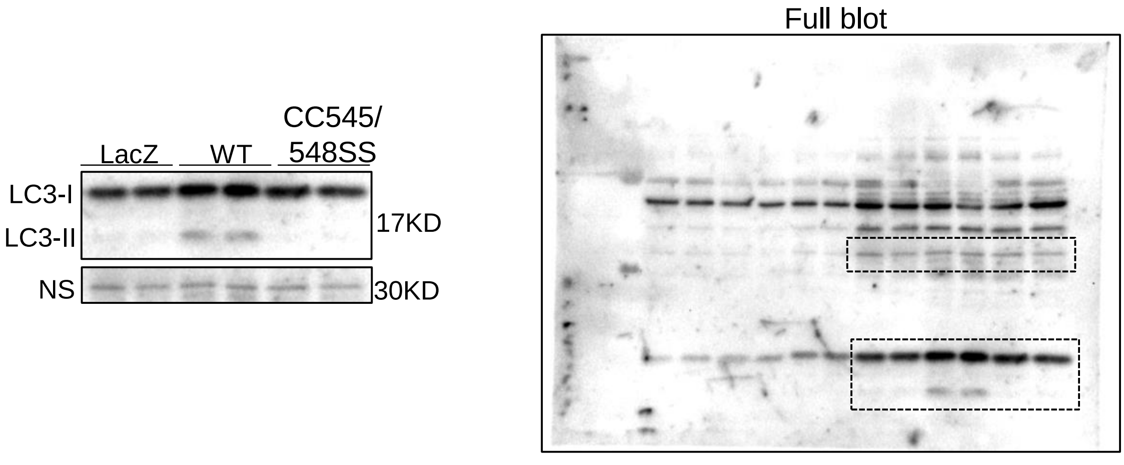

Figure 4

Figure 4A

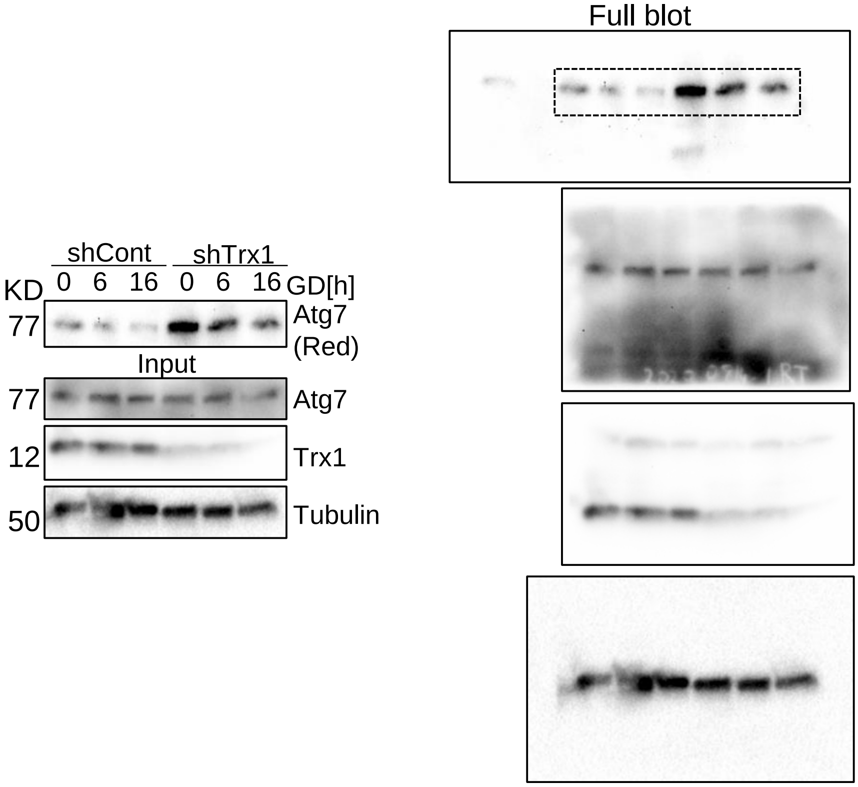

Figure 4B

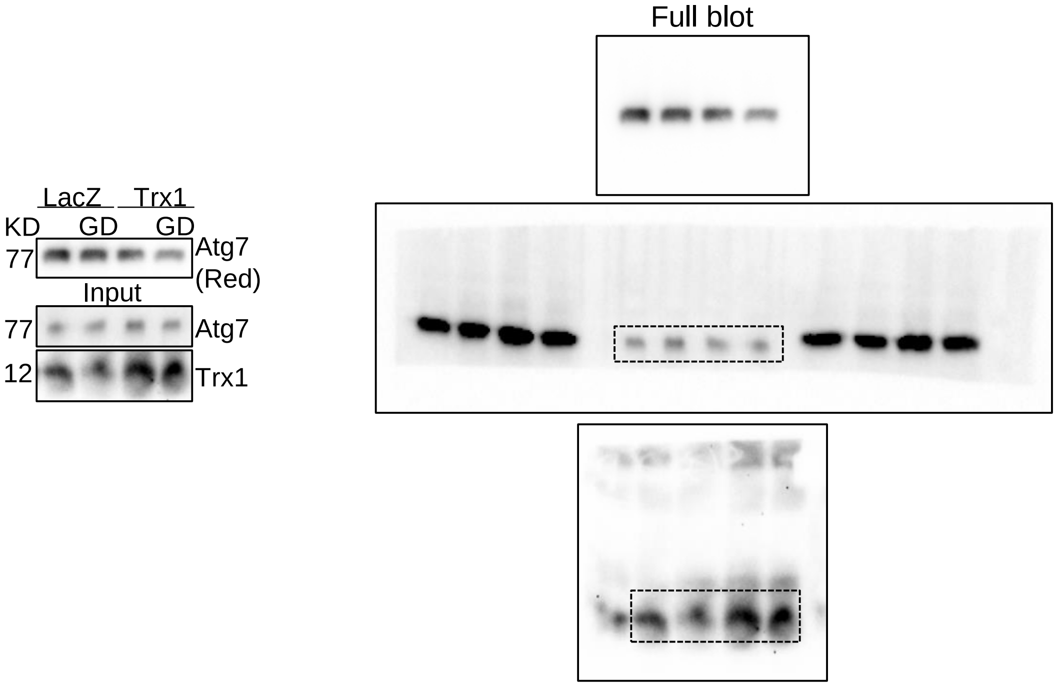

Figure 4C

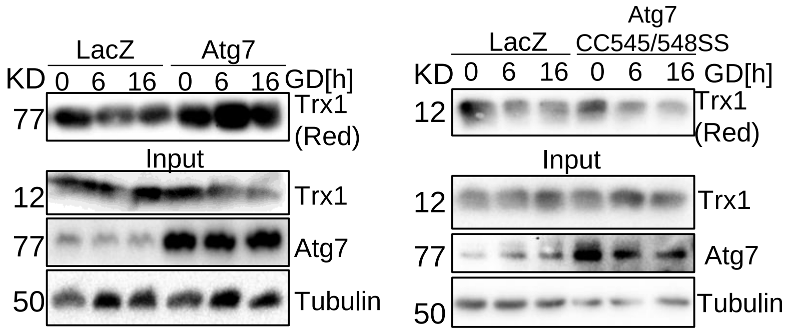

Full blot

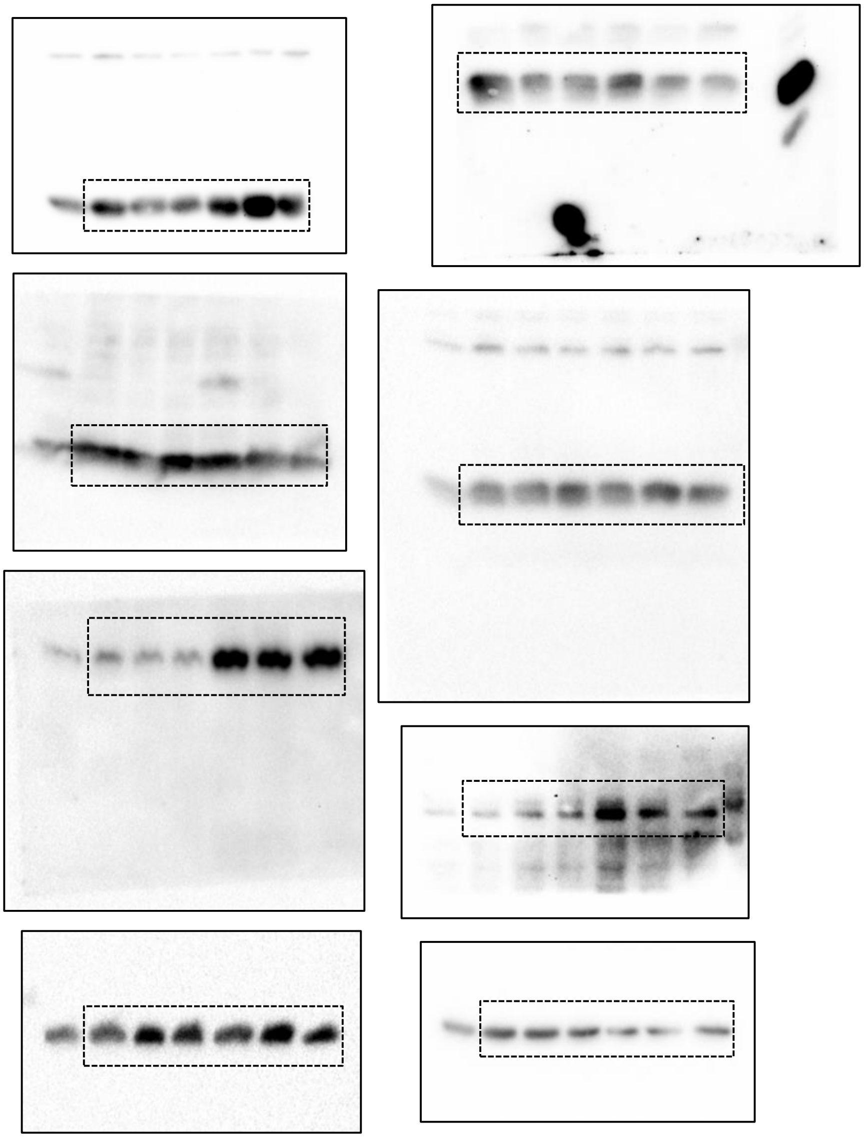

Figure 4D

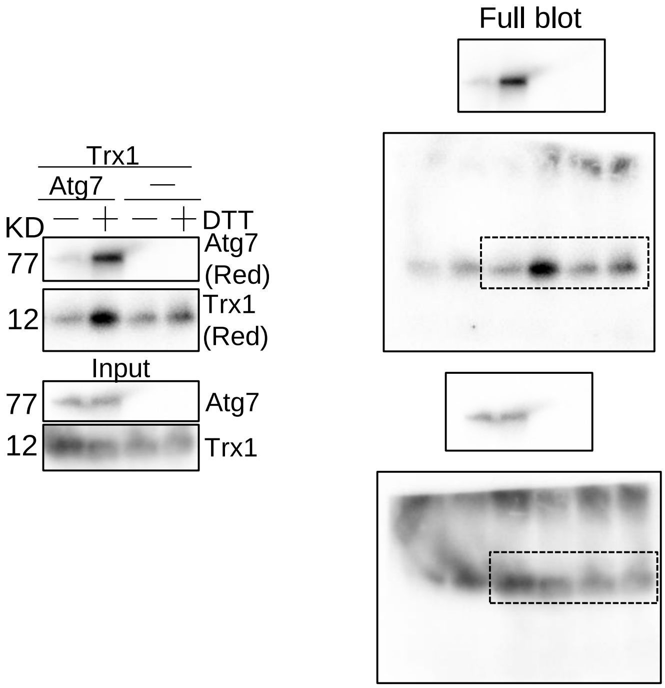

Figure 5A

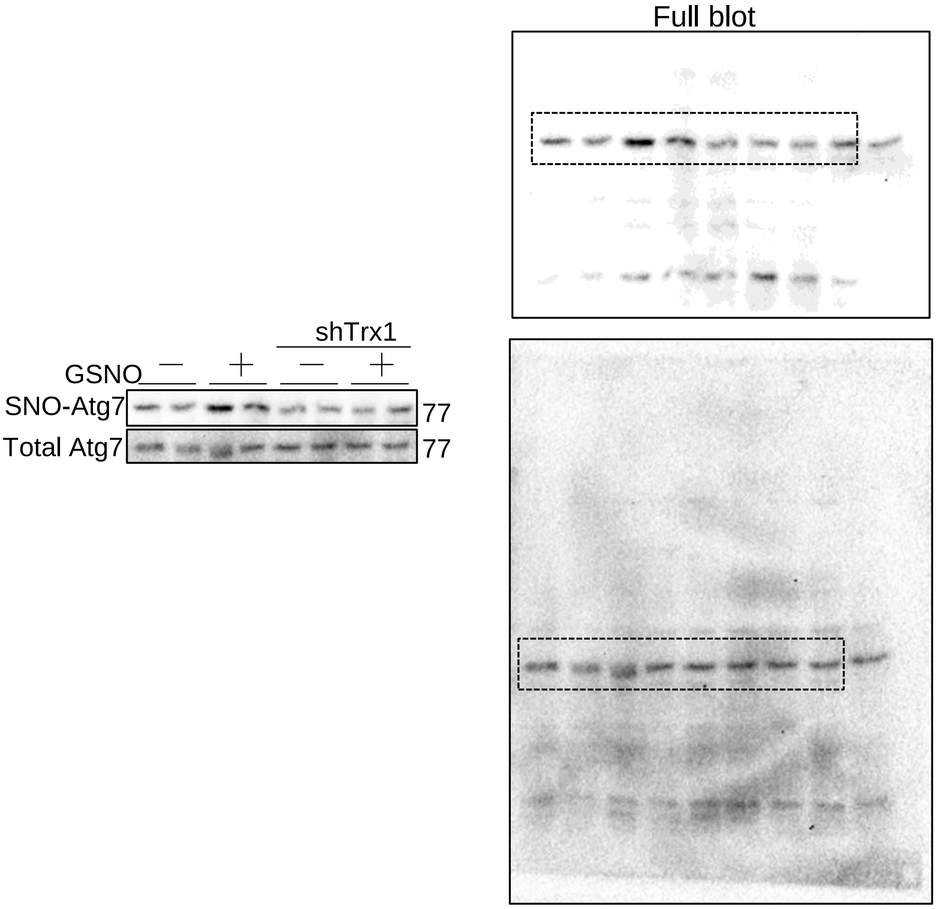

Figure 5B

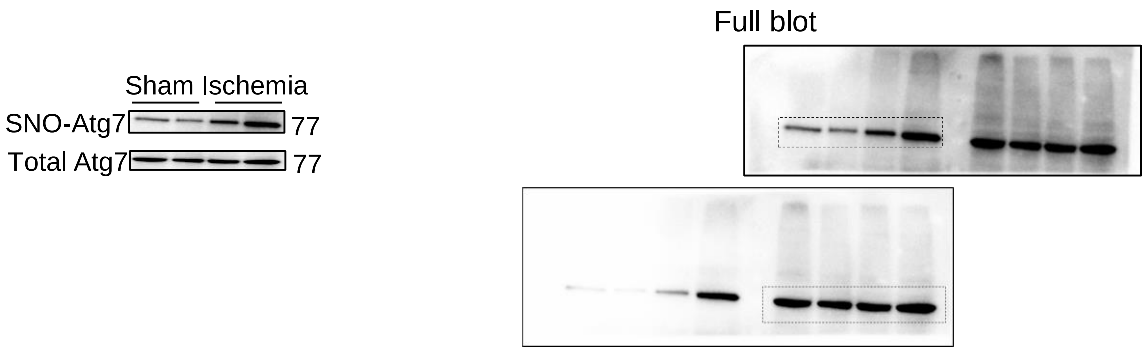

Figure 5C

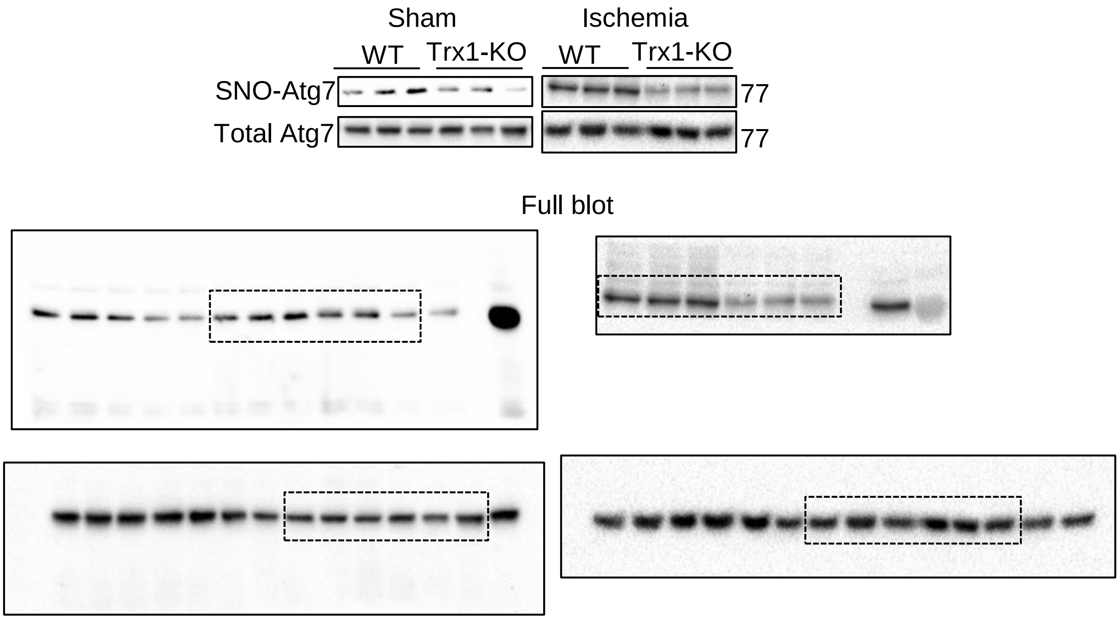

Figure 5D

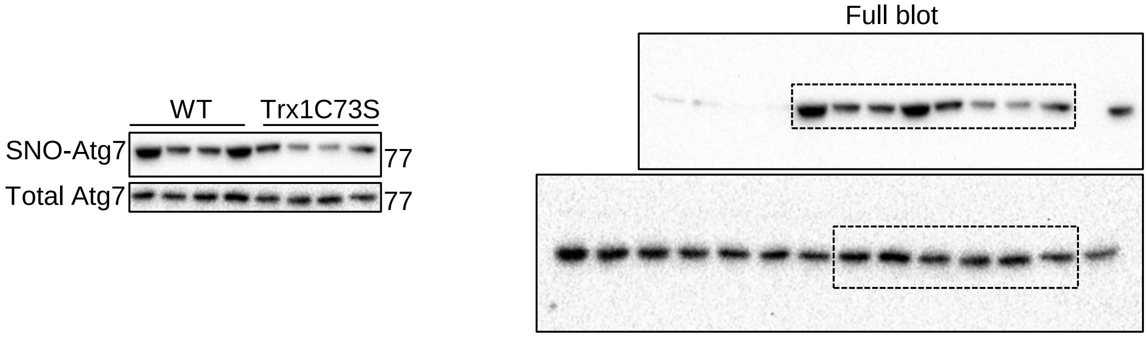

Figure 5E

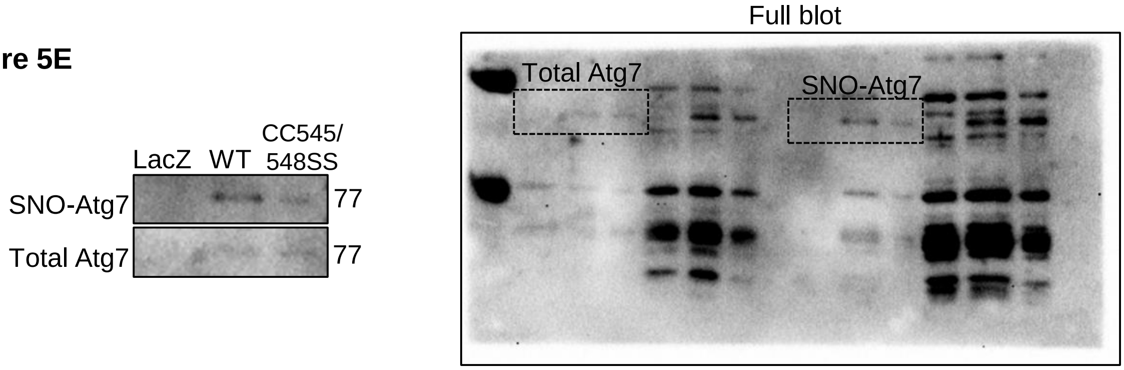

Figure 5F

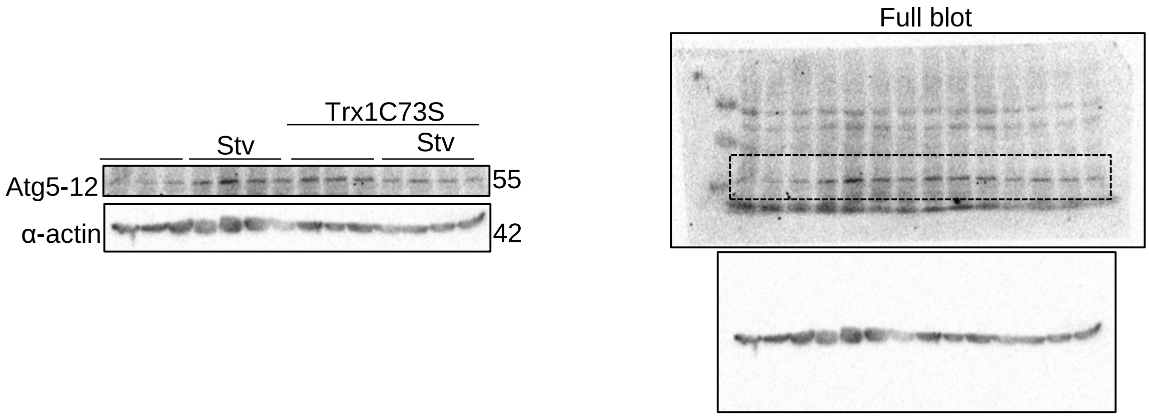

Figure 5H

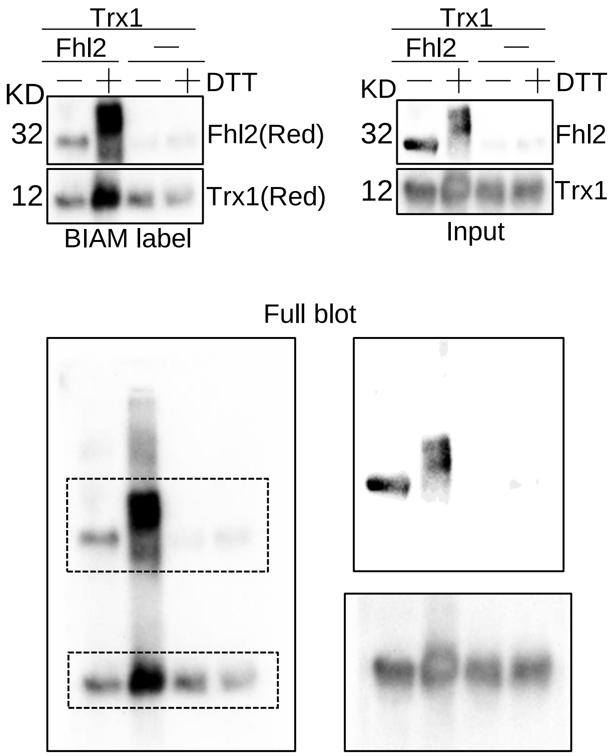

Trx1
Fhl2
—
— + — + DTT
KD
32
Fhl2(Red)
12
Trx1(Red)
BIAM label
Trx1
Fhl2
—
— + — + DTT
KD
32
Fhl2
12
Trx1
Input
Full blot

Figure 6C

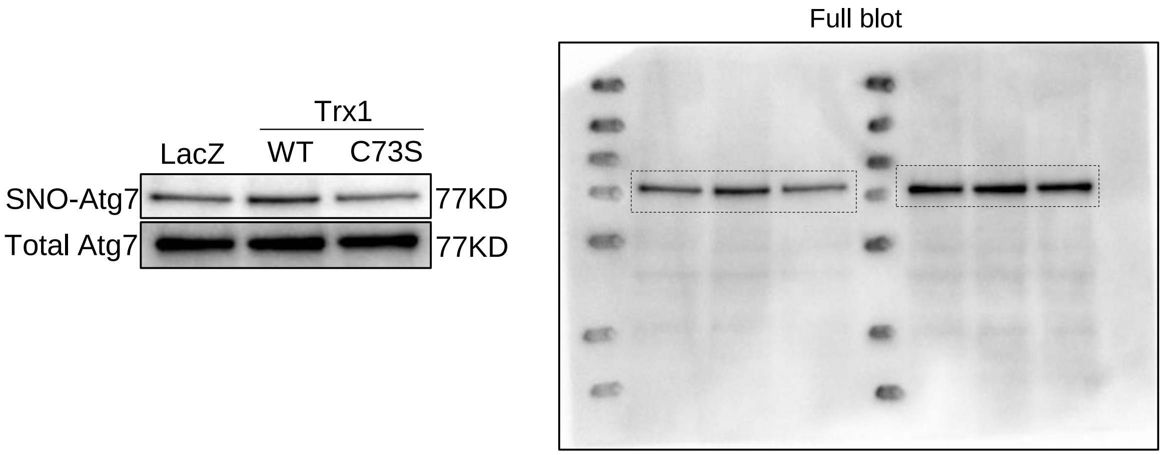

Figure 6D

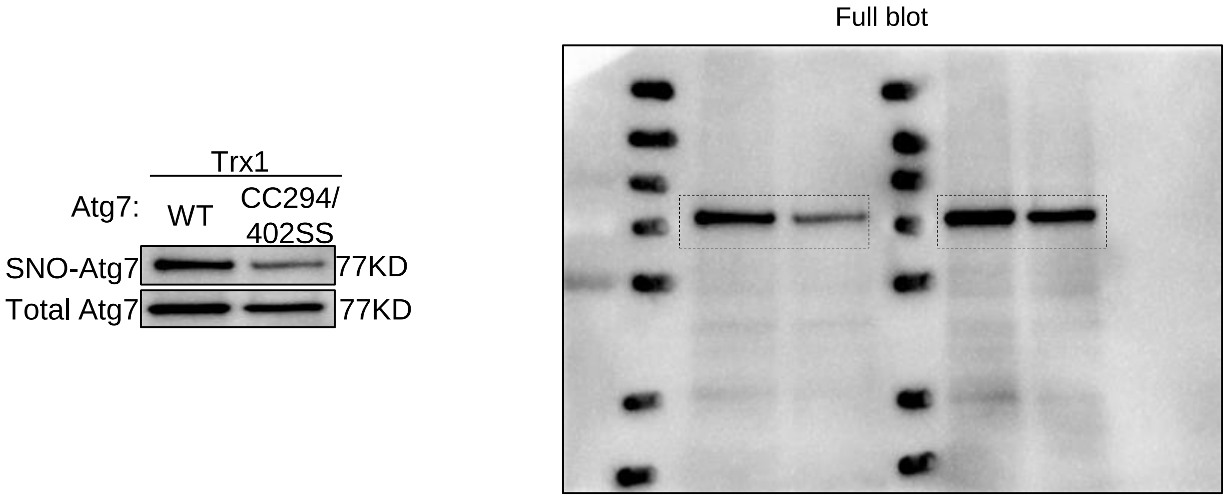

Figure 7H

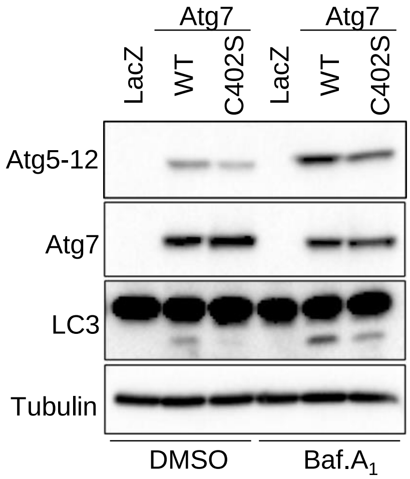
Atg7
Atg7
LacZ
WT
C402S
LacZ
WT
C402S
Atg5-12
Atg7
LC3
Tubulin
DMSO
Baf.A1

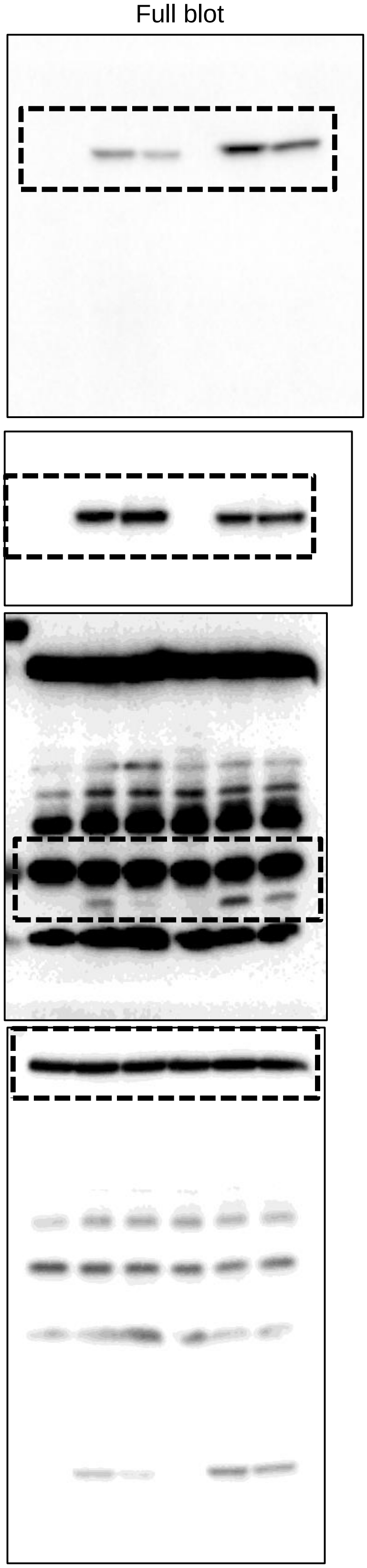
Full blot
